# MicroRNA-138 suppresses glioblastoma proliferation through downregulation of CD44

**DOI:** 10.1038/s41598-021-88615-8

**Published:** 2021-04-28

**Authors:** Margaret Yeh, Yin-Ying Wang, Ji Young Yoo, Christina Oh, Yoshihiro Otani, Jin Muk Kang, Eun S. Park, Eunhee Kim, Sangwoon Chung, Young-Jun Jeon, George A. Calin, Balveen Kaur, Zhongming Zhao, Tae Jin Lee

**Affiliations:** 1 Department of Neurosurgery, McGovern Medical School, University of Texas Health Science Center at Houston, 6431 Fannin St., MSE R117B, Houston, TX 77030 USA; 2 Center for Precision Health, School of Biomedical Informatics, University of Texas Health Science Center at Houston, 7000 Fannin St. Suite 600, Houston, TX 77030 USA; 3 Department of Biosciences, Rice University, Houston, TX USA; 4 Pulmonary, Allergy, Critical Care and Sleep Medicine, The Ohio State University Wexner Medical Center, Davis Heart and Lung Research Institute, Columbus, OH USA; 5 Department of Integrative Biotechnology, College of Biotechnology and Bioengineering, Sungkyunkwan University, Suwon, South Korea; 6 Department of Translational Molecular Pathology, Center for RNA Interference and Non-Coding RNAs, The University of Texas MD Anderson Cancer Center, Houston, TX 77030 USA

**Keywords:** CNS cancer, miRNAs

## Abstract

Tumor suppressive microRNAs (miRNAs) are increasingly implicated in the development of anti-tumor therapy by reprogramming gene network that are aberrantly regulated in cancer cells. This study aimed to determine the therapeutic potential of putative tumor suppressive miRNA, miR-138, against glioblastoma (GBM). Whole transcriptome and miRNA expression profiling analyses on human GBM patient tissues identified miR-138 as one of the significantly downregulated miRNAs with an inverse correlation with CD44 expression. Transient overexpression of miR-138 in GBM cells inhibited cell proliferation, cell cycle, migration, and wound healing capability. We unveiled that miR-138 negatively regulates the expression of CD44 by directly binding to the 3′ UTR of CD44. CD44 inhibition by miR-138 resulted in an inhibition of glioblastoma cell proliferation in vitro through cell cycle arrest as evidenced by a significant induction of p27 and its translocation into nucleus. Ectopic expression of miR-138 also increased survival rates in mice that had an intracranial xenograft tumor derived from human patient-derived primary GBM cells. In conclusion, we demonstrated a therapeutic potential of tumor suppressive miR-138 through direct downregulation of CD44 for the treatment of primary GBM.

## Introduction

The most aggressive and lethal form of brain tumors is glioblastoma (GBM)^1^. Current treatments include surgical resection, radiation, chemotherapy with temozolomide (TMZ), tumor-treating fields (TTFields) or their combination, which results in a median overall survival (OS) of around 20 months^2^. In order to prime the tumor cells in GBM patients to become responsive to current treatment options or even new therapeutics, tumor cells need to be reprogrammed by reversing the aberrantly dysregulated gene expressions across various signaling pathways. However, treatment of GBM with a promising anti-tumor agent remains as one of the most challenging tasks in pharmaceutical development, since most therapeutic agents (up to 98%) are not capable of penetrating the blood–brain barrier (BBB) to reach to the tumor site^3^. Thus, it is highly significant to develop new tumor-specific targeted therapy to improve the clinical outcomes in GBM patients with less side effects due to non-specific targeting.

MicroRNAs (miRNAs) are small noncoding RNAs, and have been implicated in the development, progression and metastasis of many types of human cancer^4^. The expression level of a certain miRNA has been used to determine whether the specific miRNA plays a tumor suppressive or oncogenic role in cancers^4^. Restoring tumor suppressive miRNAs in cancer cells has been proposed as one of potential therapeutic strategies to efficiently reprogram the aberrantly deregulated cancer networks^5^. Since downregulation of miR-138 has been reported in most type of human cancers, miR-138 has been proposed as a tumor suppressive miRNA^6–8^. In GBM, miR-138 was first reported as pro-survival onco-miR in glioma stem cells^9^. However, other studies showed that miR-138 may play tumor suppressive roles in GBM^10–14^. Although the contradictory results may originate from the heterogeneous nature of GBM tumor^15–17^, further study was needed to clarify the role and druggability of miR-138 in GBM.

For the first time, this study found from whole transcriptome and miRNA expression profiling analyses that miR-138 is downregulated in human primary GBM tissues and primary GBM cells derived from human patients with an inverse correlation with the expression of CD44. Ectopic restoration of miR-138 negatively modulated the expression of CD44 in GBM cells leading to the inhibition of cell proliferation and increased survival rate in orthotopic xenograft mouse model. We discovered that miR-138 directly regulates CD44 expression by binding to the 3′ UTR of CD44. For many years, activation of CD44 pathways has been implicated in GBM as responsible for various tumor supportive signaling pathways, such as cell motility, cell proliferation, invasion, angiogenesis and drug resistance^18^. Although specific inhibition of CD44 using antibody has been shown to decrease cancer cell proliferation, first humanized CD44-targeting monoclonal antibody developed by Roche (RO5429083) is still under clinical trials (ClinicalTrials.gov Identifiers: NCT01641250, NCT01358903)^19^. Our data strongly suggest that inhibition of CD44 signaling pathway through the restoration of miR-138 can benefit therapeutic outcomes in clinical settings for GBM patients.

## Results

### miR-138 is downregulated in glioblastoma

**Figure 1:**
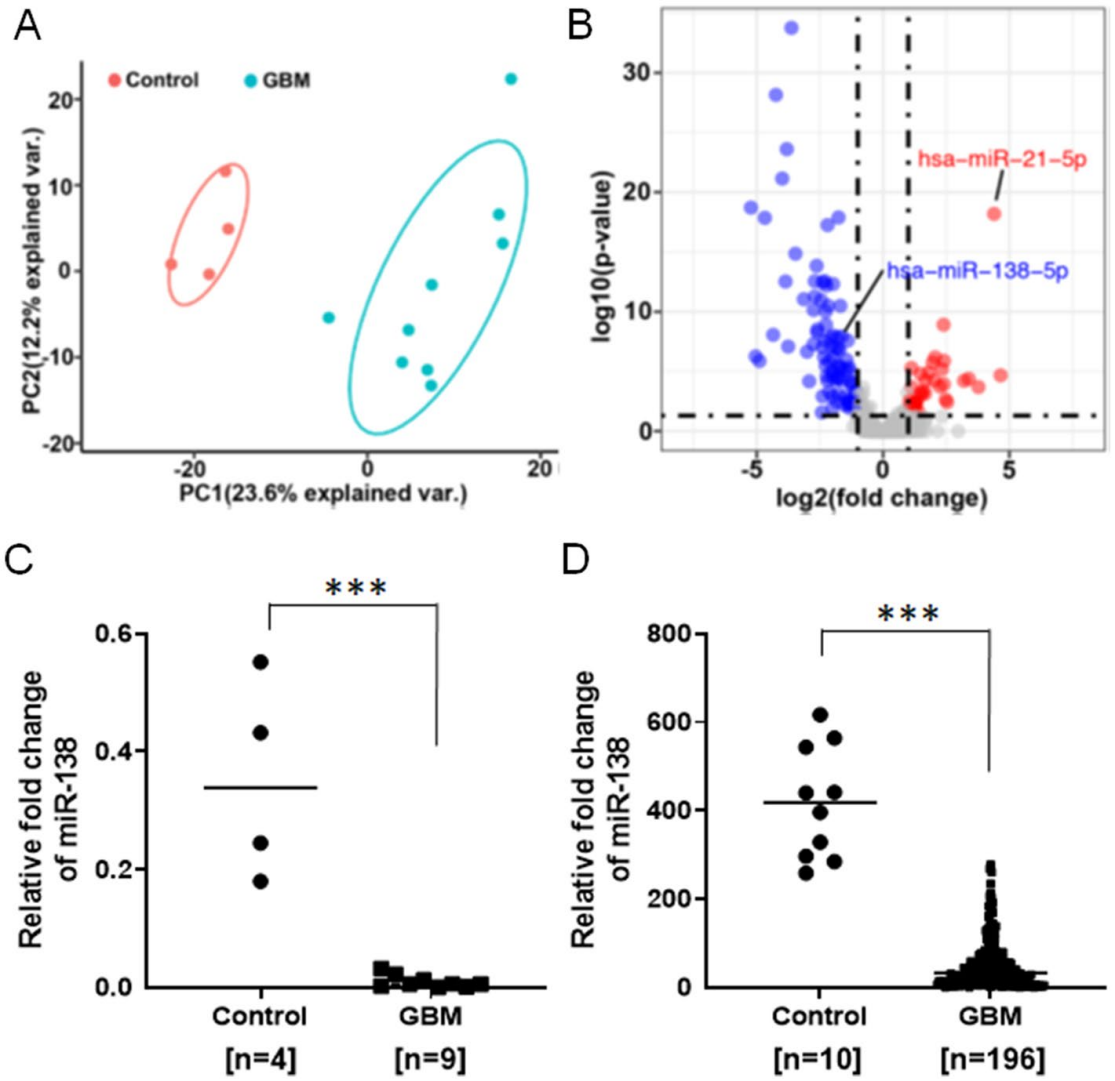
Downregulation of miR-138 in glioblastoma (GBM) patient specimens. (**A**) Principal component analyses (PCA) on miRNA expression profiling obtained by NanoString miRNA Expression Analysis from 9 GBM patients and 4 negative control samples. As a quality control, PCA plot clearly classified the samples into two groups. (**B**) Volcano plot reveals down-regulation of miR-138 in an inverse correlation with miR-21. (**C**) Expression of miR-138 in GBM samples (0.009915 ± 0.003592, n = 9) compared to negative control samples (0.3525 ± 0.08532, n = 4) analyzed by qRT-PCR using TaqMan individual miRNA assay. (**D**) The Cancer Genome Atlas Program (TCGA) database shows that miR-138 expression is downregulated in glioblastoma (GBM) compared to normal tissues. All error bars indicates standard deviations, and Student *t*-test was used to determine the significance in difference between the two groups. ***p < 0.001.

To determine whether miR-138 is tumor suppressive or tumor supportive in GBM, miRNA expression profiles were obtained by NanoString miRNA Expression Analysis from 13 frozen brain tissue samples (9 GBMs and 4 controls) (Supp Table 1). Total of 827 miRNAs were detected and the miRNA expression profiles were then subjected to principal component analysis (PCA) for quality control, which clearly classified the samples into two groups (Fig. 1A). The high expression level of miR-21 was detected from the 9 GBM samples as previously known that miR-21 is one of the most highly overexpressed miRNAs in most types of human cancer including GBM^20–22^. A total of 89 differentially expressed miRNAs (DEmiRs) were detected by using DEseq2 R package with BH adjusted p-value < 0.01 and two fold changes (Supp Table 2)^23^. Among them, miR-138 was identified as one of the down-regulated miRNAs in the GBM samples with a reverse correlation with miR-21 (Fig. 1B). Individual miRNA expression analysis by TaqMan quantitative real-time PCR (qRT-PCR) further confirmed that the relative fold change of miR-138 in the GBM patient samples was significantly down-regulated (0.009915 ± 0.003592, n = 9) when compared to negative control samples (0.3525 ± 0.08532, n = 4) (Fig. 1C, p < 0.001). We further examined the expression level of miR-138 in human patient-derived primary GBM cells, and found that the miR-138 expression was observed lower in 4 patient-derived primary GBM cells (GBM8 (0.1262 ± 0.004762), GBM12 (0.06386 ± 0.004912), GBM28 (0.07143 ± 0.004731) and GBM43 (0.08453 ± 0.002392)) than that in human neural stem cell (hNSC (1.537 ± 0.3098), a non-malignant negative control (Supp Fig. S1). The lower expression level of miR-138 was also observed in 6 glioma cell lines (LN229 (0.081 ± 0.005741), T98G (0.06172 ± 0.001572), U87 (0.07579 ± 0.002727), U373 (0.4155 ± 0.04372), LN18 (0.04435 ± 0.001073) and U251 (0.4743 ± 0.03747)) (Supp Fig. S1). From the public database, The Cancer Genome Atlas Program (TCGA) from the Betastasis (http://www.betastasis.com/glioma/tcga_gbm/), the expression of miR-138 was also found to be lower in most brain tumor types than normal brain tissues (Fig. 1D, p < 0.001). Another public database presenting single cell RNA sequencing (scRNA-Seq) profiles from 3589 single cells of GBM patient summarized 2D-tSNE representation showed that miR-138 expression is barely detectable while the expression level of miR-21 is high in neoplastic cell population (Supp Fig. S2). Taken together, our studies indicated that miR-138 is down-regulated in GBM as coincided with public databases as well as previous reports^10,14,24^, implying a possible tumor suppressive role of miR-138 in GBM tumor.

### miR-138 restoration inhibits GBM cell proliferation

**Figure 2:**
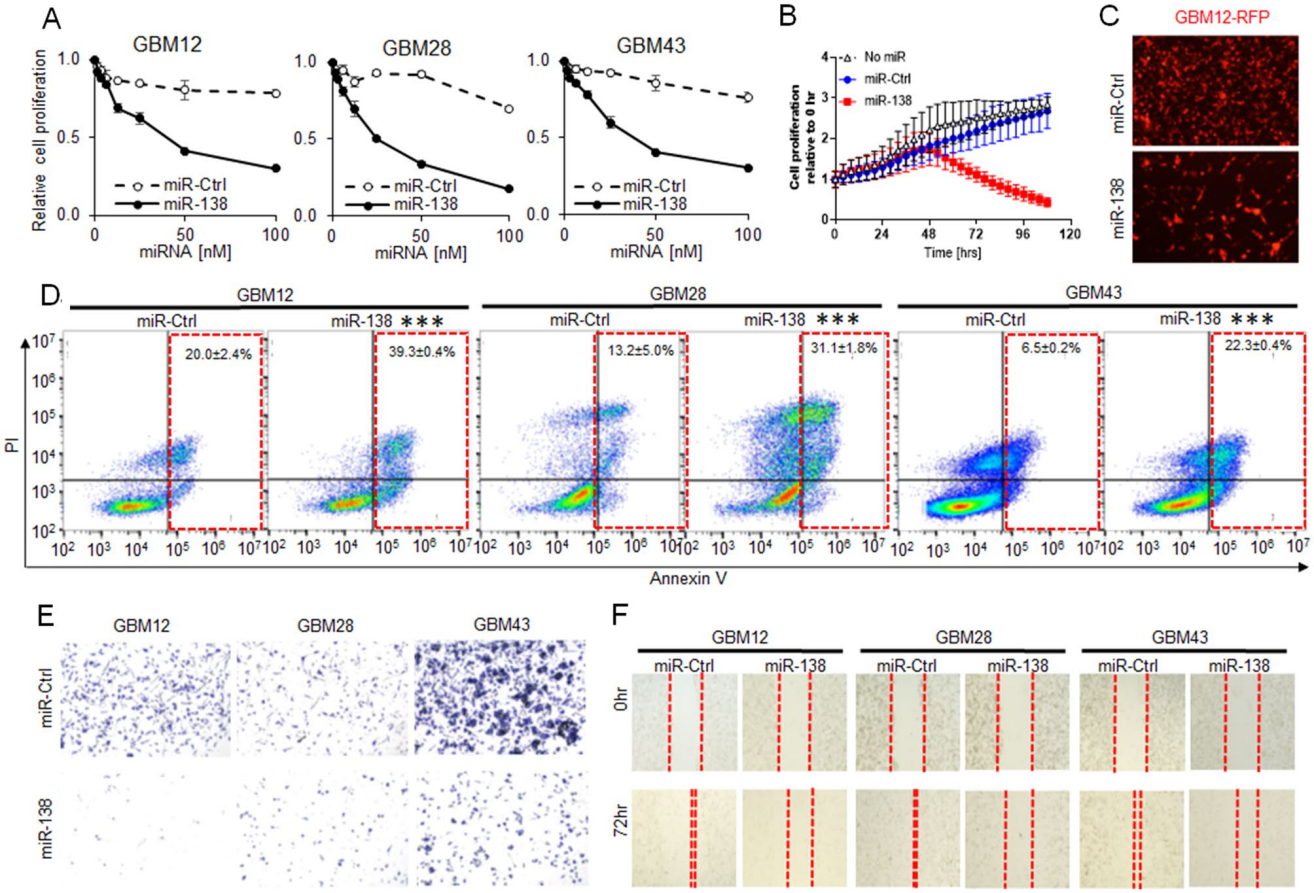
Overexpression of miR-138 inhibits cell proliferation and migration in vitro. (**A**) GBM cell proliferation with miR-138 overexpression. Patient-derived primary GBM cells (GBM12, 28 and 43) were transfected with a series of concentration of miR-138 mimics or negative control (miR-Ctrl). After 4 days of transfection, viable cells were measured by CellTiter-Glo Luminescent Cell Viability Assay. (**B**) Cell proliferation analysis by fluorescence live cell imaging every four hours on GBM12-RFP cells after transfection of 25 nM miR-138 or miR-Ctrl. (**C**) Representative fluorescence images of GBM12-RFP cells at 96 h after transfection of 25 nM miR-138 or miR-Ctrl. (**D**) Apoptosis analysis on GBM cells (GBM12, 28 and 43) after transfection of 25 nM miR-138 or miR-Ctrl followed by Annexin V-PI double staining for flow cytometry. Percentage of apoptotic cell populations were indicated in red-dotted box on each representative cytograms (n = 3). (**E**) Cell migration assay in Trans-well chamber plates. GBM cells transfected with 25 nM miR-138 or miR-Ctrl were cultured in 8 µm pore size Boyden chamber inserted into 24-well plates. The top chamber was removed 4 days later, and the migrated cells in the bottom chamber were visualized by staining with 0.5% crystal violet. Each images represent stained cells from each well obtained by bright field microscope. (**F**) Wound healing assay to evaluate the change of physical gap-closure by miR-138 overexpression. At Day 0, monolayer of GBM cells were scratched vertically followed by transfection of 25 nM miR-138 or miR-Ctrl. Representative cell images were taken at 4 days later by bright field microscope. All error bars indicates standard deviations (n = 3), and the *p*-values were determined by two-tailed student *t*-test. ***p < 0.001.

To assess a tumor suppressive potential of miR-138 in GBM, miR-138 was ectopically expressed in GBM cells to assess its impact on GBM cell proliferation. GBM patient-derived primary cells (GBM 12, 28 and 43) was transiently transfected with miR-138 mimics in comparison to scrambled negative control (miR-Ctrl). After 4 days from the transfection, all GBM cells showed a significant sensitivity to the miR-138 overexpression (Fig. 2A). The inhibition of GBM cell proliferation was dependent on the concentration of miR-138 transfected, while the concentration dependency was not observed with miR-Ctrl. Same results were also observed from 5 GBM cell lines (LN229, U87, T98G, LN18 and U373) when transiently transfected with miR-138 (Supp Fig. S3). Using GBM12-RFP cell expressing red fluorescence protein (RFP) for live cell imaging, it was shown that the proliferation of GBM cells stopped growing after 48 h post transient transfection of miR-138 mimics (Fig. 2B).

After 4 days from the miR-138 transfection, lower numbers of GBM12-RFP cells survived than the cells treated with miR-Ctrl (Fig. 2B,C). Again, the proliferation inhibition of GBM12-RFP cells was depending on the treated concentration of miR-138 (Supp Fig. S4). When the primary GBM cells (GBM12, 28 and 43) with transient overexpression of miR-138 were stained with Annexin V-PI, apoptotic cell populations were significantly increased (39.3 ± 0.4%, 31.1 ± 1.8%, 22.3 ± 0.4%, respectively) compared to the GBM cells transfected with miR-Ctrl (20.0 ± 2.4%, 13.2 ± 5.0%, 6.5 ± 0.2%, respectively) (Fig. 2D). Transient restoration of miR-138 also reduced the migration (Fig. 2E) and wound healing capability (Fig. 2F) of GBM cells. These data strongly indicated that miR-138 possesses a tumor suppressive role in GBM through an inhibition of cell proliferation.

### CD44 expression is inversely correlated with miR-138 expression in GBM

**Figure 3:**
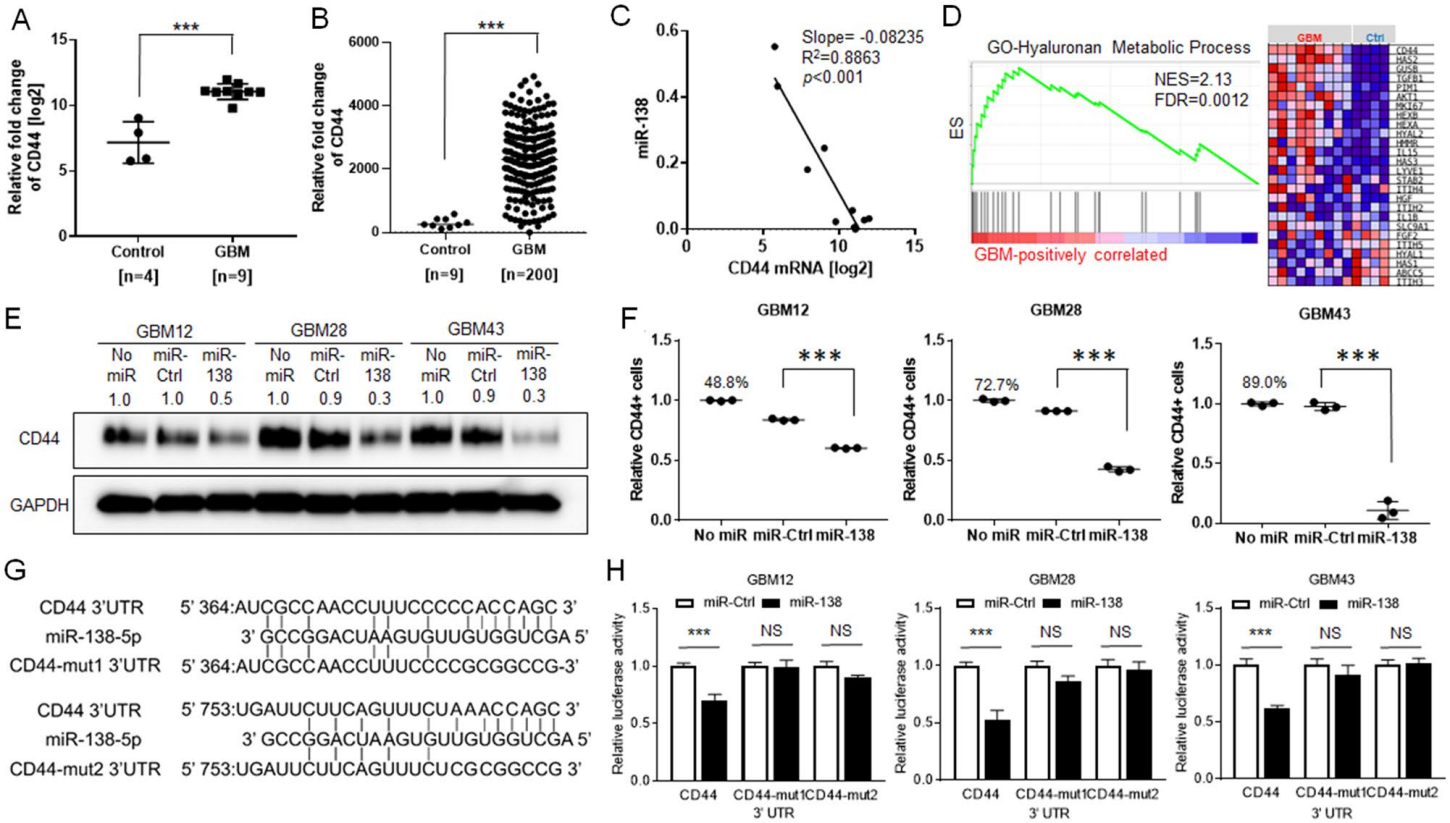
miR-138 negatively regulates the expression of *CD44* through direct targeting its 3′ UTR. (**A**) Overexpression of *CD44* in human GBM patient samples (11.06 ± 0.1995, n = 9) compared to control samples (7.168 ± 0.7913, n = 4) as measured by SyBr-Green real-time quantitative PCR (qRT-PCR) (***p < 0.001). (**B**) TCGA database showed the overexpression of CD44 in GBM (2329 ± 76.09, n = 200) compared to control samples (299.9 ± 50.01, n = 9) (***p < 0.001). (**C**) Paired expression correlation between miR-138 and CD44 in human GBM patient samples (Slope = − 0.08235, R^2^ = 0.8863, *p* < 0.001, n = 13 (9 GBM and 4 controls)). The expression levels of miR-138 and *CD44* were measured by TaqMan miRNA expression assay or SyBr-Green real-time quantitative PCR (qRT-PCR), respectively. (**D**) Enrichment plot by Gene Set Enrichment Analysis (GSEA) showed the enriched genes including CD44 involving in hyaluronan metabolic pathway. (**E**) Western blotting on GBM cells with transient overexpression of miR-138. The protein expression levels of CD44 were significantly lower than miR-Ctrl treated GBM cells. (**F**) Detection of CD44 positive cells by flow cytometry on GBM cells after miR-138 overexpression in comparison to control groups with no miR or miR-Ctrl overexpression. Transiently transfected GBM cells with miR-138 mimics were stained with FITC-labeled anti-CD44 antibodies and analyzed. FICT-negative cell population was gated by analyzing GBM cells transfected with siRNAs against CD44 (Supplementary material Fig. S5). (**G**) Schematic diagram of 3′ UTR sequences of CD44 containing two predicted miR-138 binding sites and the reporter constructs showing the mutated sequences in the seed regions (CD44-mut1 and CD44-mut2). (**H**) Luciferase reporter assays to test directing binding of miR-138 to the CD44 3′ UTR regions in GBM cells. GBM cells were transfected with luciferase reporter gene expressing DNAs containing CD44 3′ UTR, CD44-mut1 or CD44-mut2 sequences respectively. Next day, the cells were further transfected with miR-138 or miR-Ctrl for 48 h. Passively lysed cell lysates were assayed for luciferase activity by Dual Luciferase Assay kit. The relative luciferase activity values were normalized to Renilla luciferase activity as internal control. All error bars indicates standard deviations (n = 3), and the *p*-values were determined by two-tailed student *t*-test. ***p < 0.001.

Determination of direct targets of a miRNA is a key information to understand an underlying mechanism by which miR-138 plays a tumor suppressive role in GBM. To identify candidate target genes of miR-138, total RNA sequencing (RNA-seq) was performed on the 13 patient samples. Through bioinformatics analysis on the RNA-seq obtained from human gBM samples (n = 9) in comparison to those from normal (n = 4), total of 420 genes were identified as differentially expressed genes (DEGs) (Supp Table 3). Among the top 5 out of 420 DEGs, we noticed that CD44 is predicted as a significant target of miR-138 by prediction software, mirDIP (microRNA Data Integration Portal) (Supp Table 4). Individual gene expression analysis by qRT-PCR confirmed that the expression level of *CD44* is higher in GBM samples (11.06 ± 0.1995, n = 9) than that in control samples (7.168 ± 0.7913, n = 4) (Fig. 3A, p < 0.001). TCGA database also showed the *CD44* expression in GBM samples was at substantially higher level (2329 ± 76.09, n = 200) than that of control samples (299.9 ± 50.01, n = 9) (Fig. 3B, p < 0.001). Single cell-RNA sequencing (scRNA-Seq) database also showed the overexpression of *CD44* in neoplastic cell population in GBM patients (Supp Fig. S5). When plotted the expression levels of miR-138 and *CD44* in the human GBM and normal samples (n = 13), it clearly showed an inverse correlation between miR-138 and *CD44* (Fig. 3C, Slope = − 0.08235, R^2^ = 0.8863, *p* < 0.001). CD44 has been known to be overexpressed in GBM as well as many cancers^18^. As a cellular receptor of hyaluronan, the overexpression of CD44 involves in cell proliferation signaling pathway in neoplastic cells^25^. Gene Set Enrichment Analysis (GSEA) on the RNA-seq data revealed that genes involving in hyaluronan metabolic process including *CD44* are highly enriched in the GBM samples compared to control samples with the normalized enrichment score (NES) of 2.13 and false discovery rate (FDR) of 0.0012 (Fig. 3D). These results indicate the inverse correlation between miR-138 and CD44. The specific regulation between the two molecules during GBM progression requires further investigation.

### CD44 is a direct target of miR-138

The inverse expression correlation between miR-138 and *CD44* suggests that *CD44* is a possible target gene of miR-138. *CD44* overexpression and the activation of CD44 signaling pathway have been implicated in GBM with pro-survival functions^18^. Our observation on miR-138 mediated inhibition of GBM cell proliferation (Fig. 2) might be a result of CD44 pathway inhibition by miR-138. Ectopic overexpression of miR-138 reduced the protein expression of CD44 in GBM cells as observed by western blotting (Fig. 3E). Flow cytometry experiment showed that transient transfection of miR-138 reduced CD44-positive population of GBM cells (Fig. 3F and Supp Fig. S6), further supporting a negative regulation of CD44 by miR-138. Since miRNAs regulate the target gene expression through a direct binding of miRNA to the 3′ UTR of target gene mRNA, dual luciferase reporter assay was performed with a luciferase vector plasmid containing the 3′ UTR sequence of human CD44. Bioinformatics search revealed that the 3′ UTR region of human CD44 contains two putative binding sites (CD44 364–370 and CD44 753–759) matched with the seed sequence of miR-138-5p (Fig. 3G). When GBM cells were co-transfected with miR-138 mimics and the luciferase reporter vector plasmids, the luciferase activity was significantly decreased by miR-138 indicating a direct interaction between miR-138 and the 3′ UTR region of CD44 (Fig. 3H). The repression was rescued by mutations in the putative binding sites, since miR-138 did not decrease the luciferase activity of GBM cells transfected with mutated reporter vector plasmids (Fig. 3H). These data clearly demonstrated that miR-138 down-regulates the expression of *CD44* through direct binding to the 3′ UTR region of *CD44*.

### CD44 inhibition by miR-138 induces cell cycle arrest through p27 activation

**Figure 4:**
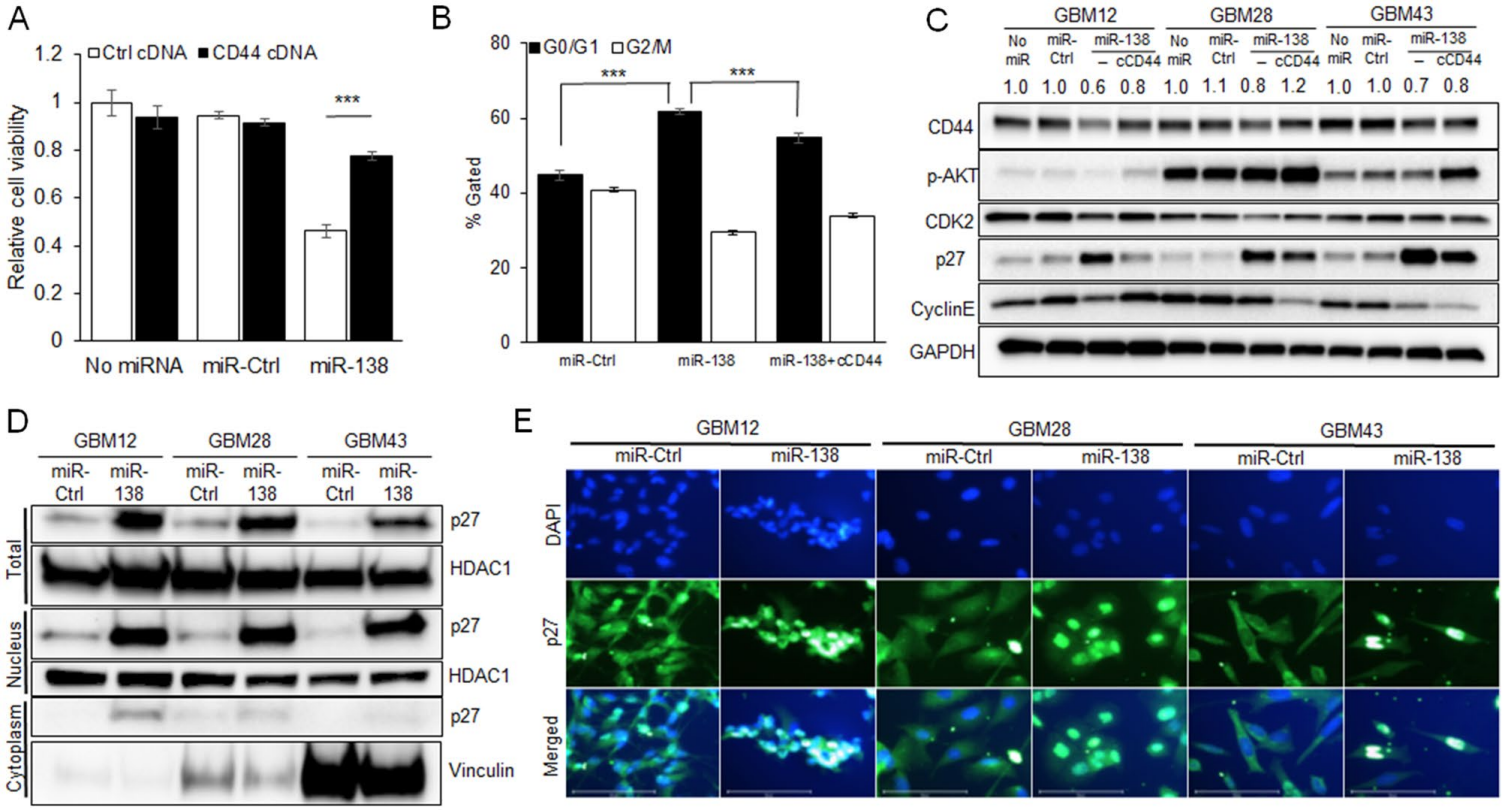
CD44 inhibition by miR-138 induces cell cycle arrest through upregulation of cell cycle inhibitor p27. (**A**) Cell viability of GBM cells decreases by miR-138 overexpression, which is partially rescued by ectopic overexpression of CD44. GBM cells were transfected with miR-138 or miR-Ctrl for 96 days in the presence CD44 expressing cDNA plasmids or empty control vector DNAs. Cell viability was measured by CellTiter-Glo Luminescent Cell Viability Assay (n = 3). (**B**) Cell cycle analysis by flow cytometry on GBM28 cells after 4 days of transient transfection of miR-138 or miR-Ctrl. Comparison of cell populations between G0/G1 and G2/M phases were expressed as %gated cells to total cell populations. Arrested cell cycle at G0/G1 phase by miR-138 was partially reversed by ectopic overexpression of CD44. Cell cycle data from GBM12 and GBM43 cells were shown in Supplementary material Fig. S7. Representative cytograms were shown in Supplementary material Fig. S8. (**C**) CD44 inhibition by miR-138 induces cell cycle arrest through Akt/p27 signaling axis. Transiently transfected GBM cell lysates with miR-138 or miR-Ctrl were analyzed by western blotting. miR-138 mediated CD44/Akt/p27 modulation was partially rescued by ectopic overexpression of CD44. (**D**) Western blotting showing activation and nuclear translocation of p27 in GBM cells after miR-138 overexpression. Total GBM cell lysates harvested after 4 days of transient transfection with miR-138 or miR-Ctrl were separated into nuclear and cytoplasmic fractions and analyzed by western blotting. HDAC1 was used as loading control for total cell and nucleus fractions, while Vinculin was used for cytoplasmic fractions. (**E**) Immunofluorescence staining of GBM cells that were transfected with miR-138 or miR-Ctrl for 4 days. Nuclear translocation of p27 was observed by overlapped fluorescence intensity of FITC-labeled anti-p27 antibody with DAPI staining. Scale bars indicate 100 µm.

To understand the working mechanism behind the miR-138 mediated inhibition of GBM cell proliferation, CD44 signaling pathway was further scrutinized. Transfection of miR-138 in GBM cells reduced cell viability, while CD44 restoration by co-transfection of *CD44*-expressing cDNA (cCD44) rescued the GBM cells from miR-138 mediated cell killing (Fig. 4A). In cell cycle analysis by flow cytometry on GMB cells after transient transfection of miR-138 mimics for 4 days, the G0/G1 cell populations were significantly increased indicating that cell cycle was arrested by miR-138 treatment in GBM28 cells (Fig. 4B), GBM12 or GBM43 cells (Supp Figs. S7 and S8). However, co-transfection of miR-138 with cCD44 reverted the cell cycle arrest by decreasing G0/G1 cell populations (Fig. 4B, and Supp Figs. S7 and S8). Among the downstream effectors of CD44 signaling pathway, the protein expression level of cell cycle inhibitor p27 was found to be seriously induced after miR-138 transfection in all tested GBM cells (GBM12, GBM28 and GBM43) (Fig. 4C). It was previously known that CD44 negatively regulates the expression and activation of p27 through AKT pathway^26^. However, restoration of CD44 expression level by co-transfection of GBM cells with cCD44 and miR-138 reversed the increase of p27 expression level through the gain of AKT activation (Fig. 4C). Since the activated p27 translocates into nucleus to inhibit cell cycles, the expression level of p27 was measured in both nuclear and cytoplasmic fractions from GBM cells. Treatment of miR-138 to GBM cells significantly increased the protein expression level of p27 in nuclear fraction, while the increase of p27 was barely observed in cytoplasmic fraction (Fig. 4D). Immunofluorescence imaging on the GBM cells clearly showed the strong accumulation of p27 after miR-138 treatment compared to miR-Ctrl (Fig. 4E). These data suggest that miR-138 can arrest GBM cell cycle by p27 activation through inhibition of CD44/AKT pathway.

### Ectopic expression of miR-138 improves survival rates in intracranial tumor bearing mice

**Figure 5:**
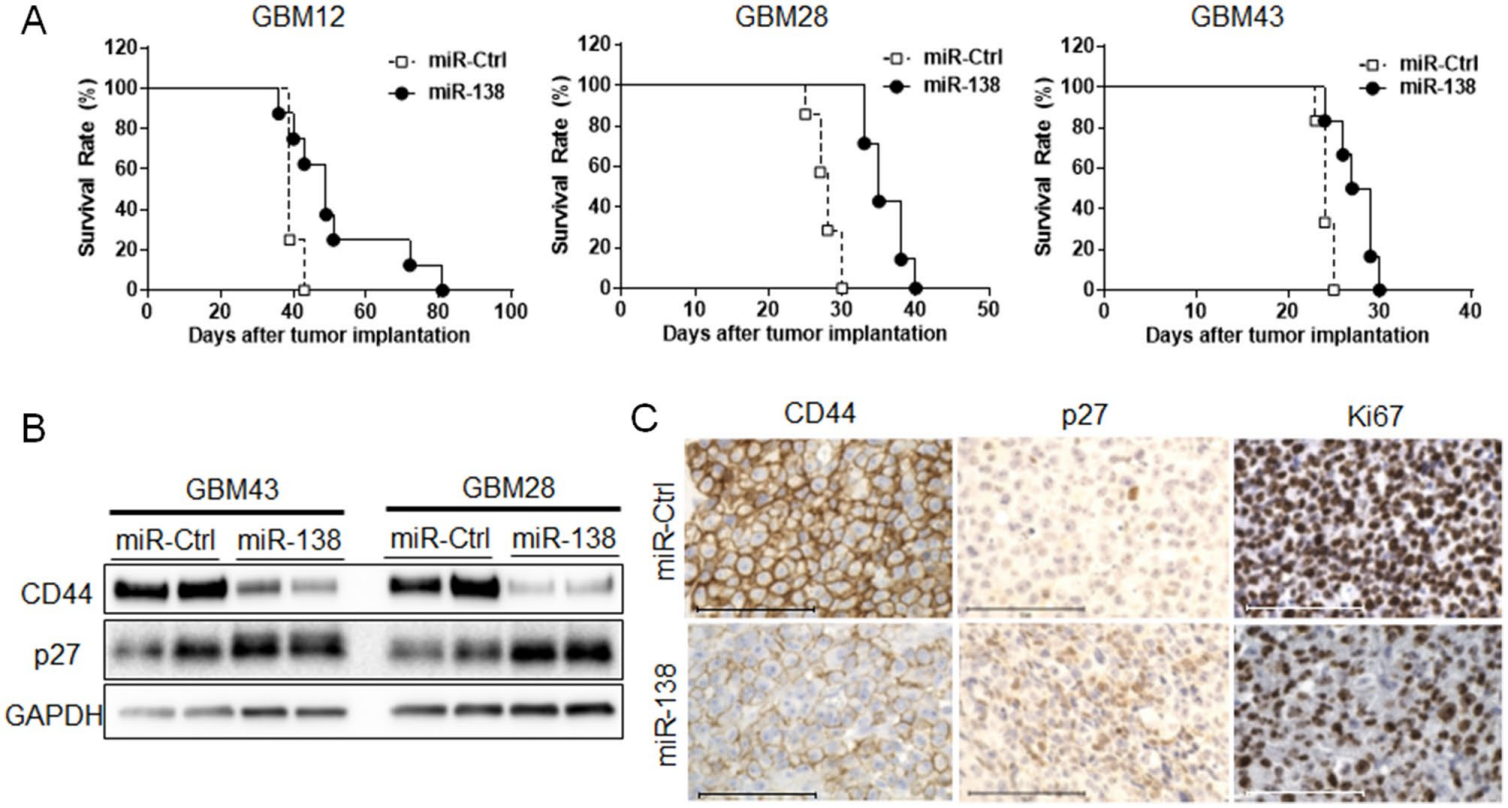
miR-138 reduces tumorigenicity of GBM cells in orthotopic in vivo model. (**A**) Mouse survival was analyzed by Kaplan–Meier survival curve. Intracranial xenograft tumor was induced by implanting GBM cells transduced with miR-138 or miR-Ctrl expressing lentiviruses. (**B**) The expression levels of CD44/p27 axis were analyzed by western blotting on the harvested mice brain tumor tissues that were derived from GBM cells (GBM28 or GBM43) with miR-138 or miR-Ctrl expression. (**C**) Representative images of tissue sections from mice brain tumor tissues after immunohistochemistry staining with anti-CD44 or anti-p27 for the change of target gene expressions by miR-138, and Ki76 for cell proliferation. Scale bars indicate 100 µm.

To evaluate the preclinical impact of miR-138 in GBM tumors, miR-138 was overexpressed in orthotopic GBM tumor xenograft. The intracranial GBM tumor was induced by injecting 2 × 10^5^ human primary GBM cells into brain of NSG mice. Kaplan–Meier survival curves showed that overall mice survival rate (n = 8) was significantly prolonged by miR-138 overexpression with a median survival compared to miR-Ctrl treated mice group (n = 8) in all tested GBM cells: GBM12 (49 vs. 39 days, *p* < 0.05), GBM28 (35 vs. 28 days, *p* < 0.001) and GBM43 (28 vs. 24 days, *p* < 0.01) (Fig. 5A). Consistent with in vitro observations as described above, the decreased CD44 and the increased p27 expressions were cleared observed from the harvested GBM tumors in western blotting (Fig. 5B). Immunohistochemistry also showed the inverse correlation between CD44 and p27 expressions in the harvested GBM tumors relative to miR-138 overexpression along with the decreased Ki67+ cells, indicating the inhibition of cell proliferation by miR-138 (Fig. 5C). Taken together, our statistically significant results suggest the conceptual therapeutic advantage of miR-138 in mice bearing an intracranial tumor.

## Discussion

Including GBM, miR-138 has been known to be downregulated from many cancers, such as melanoma, breast, neuroblastoma and pancreatic tumor^7,8^. Many studies showed that an overexpression of miR-138 can suppress cell proliferation, metastatic ability and drug resistance of cancer cells, suggesting a tumor suppressive role of miR-138. In normal brain tissues, miR-138 is highly expressed in dendrites to regulate dendritic spine morphogenesis by targeting a depalmitoylation enzyme acyl protein thioesterase 1 (APT1)^27^. Coincided with our data, low expression of miR-138 was previously observed in brain tumors and the low expression levels of miR-138 were associated with poor survival of GBM patients^10–13^. As shown in our data, a series of functional assays on the impact of miR-138 on GBM cells clearly demonstrated that the restoration of miR-138 abolishes GBM cell proliferation and migration and significantly increase survival rates of intracranial GBM tumor bearing mice. These data indicate a tumor suppressive role of miR-138 plays in primary GBM.

Concurrent transcriptome analysis on GBM patient samples has identified CD44 for the first time as a potential target of miR-138, since its expression level was also inversely correlated with that of miR-138. Further functional study data confirmed that miR-138 directly downregulates the expression of CD44 protein by binding to the 3′ UTR of CD44. Transmembrane receptor CD44 is highly overexpressed and activated not only in GBM, but also many other cancers^18^. Activation of CD44 can support tumor progression by increasing many aspects of tumor supporting signaling pathways inside the tumor cells, such as cell proliferation, cell motility, tumor growth, angiogenesis and drug resistance, and even through communication with surrounding cells within tumor microenvironment (TME)^18^. Our finding strongly indicates the therapeutic potential of miR-138 as an attractive druggable target to modulate aberrantly deregulated pro-survival gene networks in GBM cells. Clinical trials with humanized CD44-targeting monoclonal antibody are being conducted in an attempt to neutralize the activated CD44^19^. Interestingly, inhibition of CD44 by overexpressing miR-138 in GMB cells significantly increased nuclear translocation of cell cycle inhibitor p27. As a result, GBM cell population arrested at G0/G1 phase was increased in a response to the miR-138 expression. Similar observation was previously reported in acute myeloid leukemia (AML), where CD44-triggered activation of PI3K/Akt signaling pathway downregulates p27 and cytoplasmic relocation^26^. In the future studies, single cell-RNA sequencing on mouse GBM tumor after miR-138 treatment will enable to dissect the molecular changes in various type of cells within the tumor microenvironment.

A recent study has reported another aspect of miR-138 as a tumor suppressive miRNA^10^. They showed that miR-138 can educate CD4+ T cells by downregulating two immune checkpoints, programmed cell death 1 (PD-1) and cytotoxic T-lymphocyte-associated molecule 4 (CTLA-4), resulting in 43% increase in median survival time in GBM tumor bearing mice^10^. This study implied that miR-138 can induce a conversion of cold tumor to immunologically hot tumor. Future co-delivery of miR-138 into GBM cells and surrounding immunosuppressed TME cells will be an intriguing approach to demonstrate a therapeutic potential of miR-138 for GBM treatment.

In contrast, Sampath’s laboratory found that miR-138 was overexpressed in tumor-initiating glioma stem cell (GSC)^9^. They demonstrated that an ectopic expression of miR-138 promoted self-renewal potential of GSCs resulting their growth and survival. Their finding indicates that miR-138 may play a different role in a small subset of GBM stem cells, therefore further study will need to clarify whose GBM patients will benefit from the restoration of miR-138. These contradictory observations may have arisen from the heterogeneous nature of GBM^8,15–17^. Our analysis was focused on primary GBM samples without history of recurrence or previous drug treatments. In the future study, it will be intriguing to study the correlation between miR-138 and GBM subtypes (IDH WT vs IDH mutants) by DNA sequencing. Nevertheless, our results strongly demonstrated the therapeutic potential of miR-138 for primary GBM tumor, which needs to be further assessed by clinical trials. To translate our findings useful for future clinical trials, we will need to develop therapeutic strategies to deliver miR-138 efficiently into GBM cells, or even surrounding tumor-supportive immune cells. For an example, recently developed RNA-driven small RNA delivery system may be a promising candidate to achieve the challenging task since it has successfully shown its preclinical potential to specifically target brain tumor cells across BBB via folate (FA)-mediated folate receptor (FR) recognition on tumor cells^28–31^.

## Materials and methods

### Cells and cell cultures

Patient-derived de-identified primary GBM cells was kindly provided by Dr. Jann N. Sarkaria (Mayo Clinic, Rochester, MN), and their use was approved by The University of Texas Health Science Center at Houston (UTHealth, Houston) Institutional Review Board (IRB). Patient-derived primary GBM cells (GBM12, GBM28, and GBM43) were provided by Jann N. Sarkaria in Mayo Clinic, Rochester, MN, and maintained as tumor spheres in neurobasal medium supplemented with 2% B27 without vitamin A, human EGF (20 ng/mL), and basic FGF (20 ng/mL) in low-attachment cell culture flasks. Glioma cell lines (LN229, U87, U373, LN18 and T98G) were obtained from American Type Culture Collection (ATCC) and maintained in Dulbecco’s Modified Eagle’s Medium (DMEM) supplemented with 10% fetal bovine serum (FBS) and 0.1 mg/mL of penicillin/streptomycin (Penn/Strep) (Gibco BRL, Grand Island, NY). All cell cultures were maintained at 37 °C in a humidified atmosphere with 5% carbon dioxide (CO_2_). All cells were routinely monitored for morphology changes and *Mycoplasma* contamination, and authenticated by Short Tandem Repeat (STR) analysis at the University of Arizona Genetics Core. Total 22 of snap-frozen de-identified glioblastoma tumor tissues were collected in the Department of Neurosurgery at UTHealth according to a protocol approved by the UTHealth IRB.

### RNA extraction, RNA sequencing and miRNA expression profiling analysis

Total RNAs were extracted from snap-frozen GBM tissue samples using Trizol (Ambion, Thermo Fisher, USA) by following the manufacturer’s protocol. Total RNA sequencing was carried out for the extracted total RNA by Illumina HiSeq-4000 at the Advanced Technology Genomics Core (ATGC) in the University of Texas MD Anderson Cancer Center. For miRNA expression profiling, 100 ng of total RNA was processed with the Human v3 miRNA Expression Assay using NanoString nCounter system (NanoString, USA) to detect 827 human miRNAs. The miRNA profiling data were submitted to NCBI GEO (GSE165937). Expression of individual miRNA was measured by quantitative real-time PCR with TaqMan miRNA Expression kit (Applied Biosystems, Thermo Fisher Scientific, USA).

### Transcriptome analysis based on RNA-Seq

The raw sequence reads of mRNA were cleaned by trimming the low-quality bases (Q < 20), approximately 818,338,374 clean reads were generated (Supp Table 1). RNA sequencing tags were only considered when they mapped to the same DNA strand as indicated by GRCh38.p11 annotation using HISAT2^32^. After assembly, 96.53% of the total clean reads were mapped to the reference transcriptome. The fragments per kilobase of transcript per million mapped reads (FPKM) value of the transcripts was calculated based on StringTie with default parameters^32^. In the preprocessing step of mRNA analysis, non-expressed gene, which defined as having a FPKM expression level of < 1 in > 80% of samples, were removed. The remaining transcripts were then used to perform the downstream analysis. Differentially expressed genes (DEGs) between GBM and control samples were detected by Ballgown based on the expression levels obtained from StringTie with threshold of adjusted p-value less than 0.01. In addition, the differentially expressed miRNAs (DEmiRs) based on the matched mRNA-seq dataset were also detected by DEseq2 with adjusted p-value < 0.01^33^. We further required the DEGs or DEmiRs to have more than two-fold changes^34^. The functional enrichment analysis of the DEGs were conducted using the pathway databases Gene Ontology (GO) and Kyoto Encyclopedia of Genes and Genomes (KEGG) through DAVID tool (version 6.8, http://david.ncifcrf.gov). To report reliable results, the pathways and GO terms with adjusted p-value < 0.05 were selected (adjusted by Benjamin-Hochberg Procedure, false discovery rate (FDR)). The mRNA-seq data were submitted to NCBI GEO database (GSE165286).

### Ectopic expression of miRNAs

GBM cells were plated in 6-well plates at 5 × 10^5^ cells per well in DMEM containing 2% FBS without antibiotics. For transient overexpression for in vitro studies, the cells were transfected with miR-138 mimics (Dharmacon miRIDIAN microRNA Mimics, Horizon Discovery, USA) or negative control mimics after pre-incubated with Lipofectamine RNAiMAX transfection reagent (Invitrogen, Thermo Fisher Scientific, USA) as described in the manufacturer’s manual. Then the cells were incubated at 37 °C in a humidified atmosphere with 5% carbon dioxide (CO2). For in vivo studies, GBM cells were stably transduced with Human pre-miRNA Expression Lenti-miR Vector containing the full-length miR-138 and green fluorescent protein (GFP) gene (SBI, System Biosciences, USA). Human pre-miRNA Scramble Negative Control Expression Lenti Vector plasmid was used as a control. Pre-miR-138 and negative control vector plasmids were packaged with pPACKH1 Lentivector Packaging Kit (SBI, System Biosciences, USA) in HEK293TN packaging cells according to the manufacturer’s manual. After transduction, the GBM cells were sorted by FACS analysis (FACSCalibur, BD Biosciences, USA) to select GFP positive cell population containing pre-miRNA. The expression of miR-138 in the sorted cells was further confirmed by quantitative real-time PCR with TaqMan miRNA Expression kit (Applied Biosystems, Thermo Fisher Scientific, USA).

### Apoptosis and cell cycle analysis

GBM cells were transfected with miRNAs as described above in six well plates. Four days later, the cells were stained with FITC-Annexin V/PI Apoptosis Detection Kit (BD Pharmingen, USA) by following the manufacturer’s instruction, and analyzed by Beckman CytoFLEX Flow Cytometer (Beckman Coulter, USA). Annexin V-positive cells were considered to be apoptotic. For cell cycle analysis, the cells were fixed with 70% ethanol after 2 days from miRNA and stained with propidium iodide (PI) solution (Invitrogene, Thermo Fisher Sicentifics, USA) and RNase (Promega, USA) at room temperature. The PI-stained cells were subjected to flow cytometry by Beckman CytoFLEX Flow Cytometer (Beckman Coulter, USA). The obtained flow cytometry data was analyzed with FlowJo version 10 software (BD Biosciences, USA).

### Flow cytometry

Detection of CD44 positive cells was determined by flow cytometry. GBM cells transfected with miRNAs as described above in six well plates were washed with PBS 4 days after the transfection and harvested with mild trypsiniation. The harvesed cells were stained with human CD44 antibody labeled with FITC, then subjected to flow cytometry by Beckman CytoFLEX Flow Cytometer (Beckman Coulter, USA). The obtained flow cytometry data was analyzed to determine the population of CD44 positive GBM cells against non-FITC cell population using FlowJo version 10 software (BD Biosciences, USA).

### Cell proliferation assay

GBM cells plated in 96 well plates were transfected with miRNAs as described above. Four days later, each wells were treated with equal volume of CellTiter-Glo Luminescent Cell Viability Assay. After 10 min of incubation, the luminescence intensity was measured by Synergy H1 multi-mode microplate reader (BioTek Instruments, USA). When GBM12-RFP cell was used, the end-point cell viability was imaged by visualizing RFP-positive cells with excitation 532 nm and emission 588 nm using EVOS FL Auto2 Cell Imaging System (Thermo Fisher Scientific, USA).

### Cell migration assay

GBM cells were transiently transfected with miRNAs as described above in a 8 µm pore size Boyden chamber (Transwell from Corning Costar, USA), which was then inserted into 24 well plates. Four days later, the migrated cells in the bottom chamber were fixed with 1% glutaraldehyde followed by staining with 0.5% crystal violet. Images of stained cells from each well were obtained by bright field microscope (EVOS FL Auto2, Thermo Fisher Scientific, USA).

### Wound healing assay

GBM cells were plated in 12 well plates. Next day, the cell layer was scratched vertically using a sterile micropipette tip. Detached cells were washed off with PBS, and the wells from each group were imaged under a bright field microscope (EVOS FL Auto2, Thermo Fisher Scientific, USA) as Day 0. The adhered cells were then transiently transfected with miRNAs as decribed above. Four days later, each well was imaged.

### Western blotting

GBM cells from in vitro studies were washed with cold PBS for three times, and harvested with RIPA buffer (Pierce, USA). Cell lysates were fractionated by 4–20% Criterion TGX SDS-PAGE Pre-cast gels (Bio-Rad, USA), then transferred to 0.45 µm Amersham Protran nitrocellulose (NC) membranes (Cytiva, USA). After blocking with 5% BSA-containing TBS blocking buffer, the NC membranes were incubated with primary antibodies against cleaved PARP (Cell Signaling Technology, Waltham, MA, USA); BAI1 and GAPDH (Abcam, Cambridge, MA, USA) followed by HRP-conjugated secondary anti-mouse antibody (GE Healthcare, Piscataway, NJ, USA), and HRP-conjugated secondary goat anti-rabbit antibody (Dako, Hamburg, Germany). All antibodies were diluted 1:1000 and the immunoreactive bands were visualized with enhanced chemiluminescence (ECL) (GE Healthcare, Piscataway, NJ, USA) using ChemiDoc MP (Bio-Rad, Hercules, CA, USA).

### Dual luciferase reporter assay

Firefly/Renilla Duo-Luciferase reporter vector containing the 3′ UTR clone of human CD44 was purchased from GeneCopoeia, USA. The 3′ UTR of human CD44 was predicted to include two binding sites of miR-138 at 364 and 753 bases, respectively. Two mutations (CD44-mut1 starting at base 364 and CD44-mut2 starting at base 753) were introduced into the miR-138 binding sites by replacing adenines and guanosines within the seed sequences to cytosines using site-directed mutagenesis (GenScript, USA). The plasimd DNAs amplified from E.coli were prepared by Maxi kit (Qiagen, USA). GBM cells were plated in 12 well plates 1 day before transfection of 1 μg CD44 3′ UTR DNA, CD44-mut1 or CD44-mut2 with Lipofectamine 3000 (Invitrogen, Thermo Fisher, USA). Next day, the cells were transfected with miRNAs as described above. After further 48 h, cells were lysed with Passive Lysis Buffer (Promega, USA) and assayed with Dual Luciferase Assay kit (Promega, USA) according to the manufacturer’s instruction.

### In vivo studies

Six to eight weeks old NSG mice (NOD-scid IL2Rgamma^null^) purchased from the Jackson Laboratory, USA were housed and handled in accordance with the guideline of UTHealth Center for Laboratory Animal Medicine and Care (CLAMC) and the animal protocols approved by the UTHealth Animal Welfare Committee (AWC). All animal studies were carried out in compliance with the ARRIVE guidelines (http://www.nc3rs.org.uk/page.asp?id=1357). Intracranial glioblastoma xenograft was generated in the NSG mice by implanting GBM cells as previously described^28,29^.

Survival studies used eight mice per each groups. Tumor tissues were harvested from the mice brain, then either snap frozen in liquid nitrogen for western blotting analysis or fixed in 4% PBS-saturated formaldehyde. The fixed tissues were embedded in paraffin and sectioned at 5 µm thickness. Representative sections from each group were stained with Hematoxylin and Eosin (H&E) to be examined under bright field microscope (EVOS FL Auto2, Thermo Fisher Scientific, USA). For immunohistochemistry (IHC), sectioned tissue slides were stained with CD44, p27 or Ki67 antibodies (Cell Signaling, USA).

### Statistical analysis

All statistical analysis was carried out with GraphPad Prism 7 software (GraphPad, USA). Data were analyzed using unpaired, two-tailed *t*-tests when comparing the difference in two groups. ANOVA with Tukey’s post-test was used to compare data in experiments to compare more than two variables. Kaplan–Meier survival curves (n = 8) were compared by using the log-rank test. Statistical significance was determined by *p*-value lower than 0.05. Multiple test correction was adjusted by Benjamini-Hochberg (BH) procedure^23^. Data variations in a group were expressed as mean ± SD (standard deviation).

## Supplementary Information

Supplementary Information.
