# Supplementary material for: MicroRNA-138 suppresses glioblastoma proliferation through downregulation of CD44
Source: Sci Rep. 2021 Apr 28;11:9219. doi: 10.1038/s41598-021-88615-8 (PMC8080729; doi:10.1038/s41598-021-88615-8)
Supplement: Supplementary file 1 — Supplementary Information. [file 41598_2021_88615_MOESM1_ESM.docx]

MicroRNA-138 Suppresses Glioblastoma Proliferation through Downregulation of CD44

Margaret Yeh ^1,†^, Yinying Wang ^2,†^, Ji Young Yoo ^1^, Christina Oh ^3^, Yoshihiro Otani ^1^, Jin Muk Kang ^1^, Eun S. Park ^1^, Eunhee Kim ^1^, Sangwoon Chung ^4^,Young-Jun Jeon ^5^, George A. Calin ^6^, Balveen Kaur ^1^, Zhongming Zhao ^2,^* and Tae Jin Lee ^1,^*

^1^Department of Neurosurgery, University of Texas Health Science Center at Houston, Houston, TX;

^2^Center for Precision Health, School of Biomedical Informatics, University of Texas Health Science Center at Houston, Houston, TX;

^3^Department of Biosciences, Rice University, Houston, TX;

^4^Pulmonary, Allergy, Critical Care and Sleep Medicine, The Ohio State University Wexner Medical Center, Davis Heart and Lung Research Institute, Columbus, OH;

^5^Department of Integrative Biotechnology, College of Biotechnology and Bioengineering, Sungkyunkwan University, Suwon, South Korea;

^6^Department of Experimental Therapeutics, Center for RNA Interference and Non-Coding RNAs, The University of Texas MD Anderson Cancer Center, Houston, TX;

†M.Y. and Y.W. contributed equally to this work.

***Corresponding Author:**

Dr. Tae Jin Lee, Department of Neurosurgery, University of Texas Health Science Center at Houston, 6431 Fannin St., MSE R117B, Houston, TX, 77030 ([Tae.Jin.Lee@uth.tmc.edu](javascript:void(0);));

Dr. Zhongming Zhao, Center for Precision Health, School of Biomedical Informatics, The University of Texas Health Science Center at Houston, 7000 Fannin St. Suite 600, Houston, TX 77030, USA (Zhongming.Zhao@uth.tmc.edu)

**Running Title:** MiR-138 suppresses glioblastoma through CD44.

**Supplementary Tables**

**
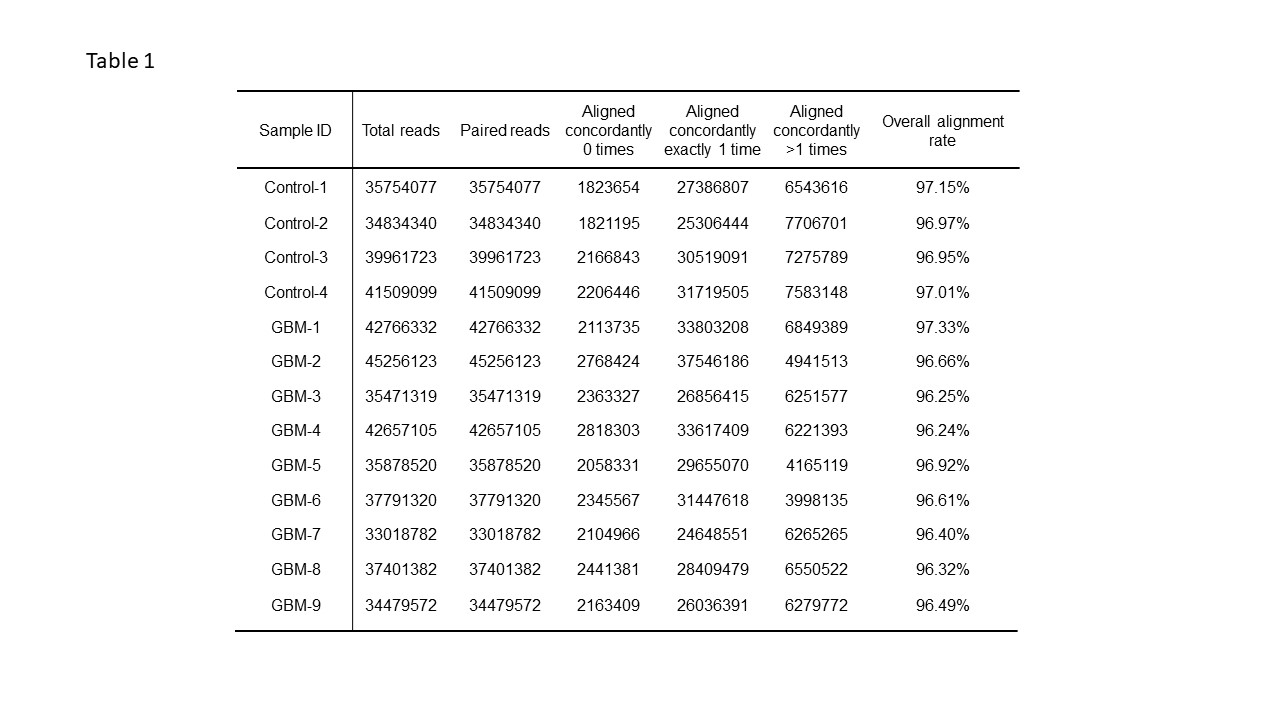
**

**Supp Table 1.** Alignment rates of RNA sequencing based on HISAT2. The raw sequence reads of mRNA were cleaned by trimming the low-quality bases (Q < 20), and approximately 496,779,694 clean reads were generated. RNA sequencing tags were only considered when they are mapped to the same DNA strand as indicated by GRCh38.p11 annotation using HISAT2.

| **miRNA** | **log2FC** | **Adjusted *p* value** |
| --- | --- | --- |
| hsa-miR-504-5p | -3.63 | 1.75E-34 |
| hsa-miR-128-3p | -4.24 | 7.10E-29 |
| hsa-miR-139-5p | -3.81 | 2.46E-24 |
| hsa-miR-139-3p | -3.99 | 7.14E-22 |
| hsa-miR-137 | -5.23 | 1.94E-19 |
| hsa-miR-107 | -1.76 | 1.29E-18 |
| hsa-miR-218-5p | -4.68 | 1.39E-18 |
| hsa-miR-1249-3p | -2.19 | 5.58E-18 |
| hsa-miR-487b-3p | -3.47 | 1.40E-15 |
| hsa-miR-4443 | -2.63 | 1.44E-14 |
| hsa-miR-628-5p | -2.70 | 2.78E-13 |
| hsa-miR-330-3p | -2.30 | 2.78E-13 |
| hsa-miR-433-3p | -3.86 | 2.96E-13 |
| hsa-miR-329-3p | -2.39 | 3.42E-13 |
| hsa-miR-1185-5p | -1.98 | 4.84E-13 |
| hsa-miR-889-3p | -2.25 | 6.59E-13 |
| hsa-miR-874-3p | -2.70 | 5.20E-12 |
| hsa-miR-323a-3p | -3.14 | 8.94E-12 |
| hsa-miR-410-3p | -2.45 | 1.10E-11 |
| hsa-miR-769-3p | -2.17 | 3.22E-11 |
| hsa-miR-1197 | -1.69 | 3.22E-11 |
| hsa-miR-769-5p | -2.75 | 7.09E-11 |
| hsa-miR-381-3p | -2.27 | 9.42E-11 |
| hsa-miR-873-5p | -2.26 | 1.23E-09 |
| hsa-miR-656-3p | -2.60 | 3.04E-09 |
| hsa-miR-582-5p | -2.61 | 5.31E-09 |
| hsa-miR-129-5p | -4.35 | 8.66E-09 |
| hsa-miR-328-3p | -1.97 | 9.73E-09 |
| hsa-miR-381-5p | -1.63 | 1.17E-08 |
| hsa-miR-29c-3p | -1.79 | 1.41E-08 |
| hsa-miR-138-5p | -2.09 | 1.52E-08 |
| hsa-miR-324-5p | -1.40 | 2.60E-08 |
| hsa-miR-379-5p | -2.30 | 2.77E-08 |
| hsa-miR-485-3p | -2.75 | 5.14E-08 |
| hsa-miR-132-3p | -2.05 | 7.08E-08 |
| hsa-miR-1290 | 3.20 | 6.07E-05 |
| hsa-miR-493-3p | 1.68 | 5.74E-05 |
| hsa-miR-10a-5p | 3.39 | 4.11E-05 |
| hsa-miR-1246 | 4.65 | 2.12E-05 |
| hsa-miR-15b-5p | 1.49 | 1.61E-05 |
| hsa-miR-4516 | 1.81 | 1.22E-05 |
| hsa-miR-142-3p | 2.33 | 6.55E-06 |
| hsa-miR-15a-5p | 1.12 | 5.09E-06 |
| hsa-miR-135b-5p | 1.96 | 1.76E-06 |
| hsa-miR-574-5p | 2.41 | 1.30E-06 |
| hsa-miR-210-3p | 2.06 | 6.54E-07 |
| hsa-miR-155-5p | 2.39 | 1.23E-09 |
| hsa-miR-21-5p | 4.39 | 6.49E-19 |

**Supp Table 2.** Top most differently expressed miRNAs (DEmiRs) from miRNA expression assay on human GBM patient samples analyzed by NanoString nCounter system. The expression level changes of DEmiRs in GBM versus control samples were expressed as the absolute log2-transformed fold change (log2FC).

| **Gene** | **LogFC** | **Adjusted *p* value** |
| --- | --- | --- |
| HIST1H3F | 5.83 | 1.19E-03 |
| HIST1H2BH | 5.73 | 3.98E-03 |
| TNC | 5.67 | 7.49E-03 |
| CD44 | 5.04 | 3.15E-03 |
| POLA1 | 4.70 | 7.58E-03 |
| ABCC3 | 4.68 | 3.84E-03 |
| ADAMTS9 | 4.66 | 7.31E-03 |
| MRC2 | 4.39 | 1.78E-03 |
| PLCB3 | 4.19 | 6.04E-04 |
| TOP2A | 4.08 | 7.44E-03 |
| HIST1H2AH | 4.03 | 6.27E-03 |
| HIST1H2AI | 4.00 | 2.15E-03 |
| CENPF | 3.66 | 8.24E-03 |
| HIST2H3C | 3.59 | 7.57E-03 |
| COL4A1 | 3.49 | 3.24E-04 |
| PTGFRN | 3.45 | 4.16E-03 |
| SBNO2 | 3.44 | 4.17E-03 |
| HIST1H3G | 3.36 | 6.88E-03 |
| C15orf52 | 3.21 | 1.71E-03 |
| MMP14 | 3.16 | 8.93E-03 |
| LRRC7 | -5.25 | 2.81E-05 |
| LRFN5 | -5.31 | 3.90E-07 |
| GABRB3 | -5.31 | 3.90E-07 |
| CABP1 | -5.45 | 1.25E-03 |
| DLGAP3 | -5.47 | 1.19E-03 |
| GRM5 | -5.56 | 1.19E-03 |
| NRGN | -5.63 | 1.50E-03 |
| SV2B | -5.79 | 1.18E-04 |
| SLC12A5 | -5.81 | 4.37E-03 |
| CREG2 | -5.86 | 2.32E-03 |
| GRIN1 | -5.86 | 1.47E-04 |
| PLCH2 | -5.94 | 9.77E-07 |
| HCN1 | -6.16 | 4.09E-04 |
| NEFL | -6.27 | 3.98E-03 |
| CAMK2A | -6.35 | 5.83E-03 |
| SNCG | -6.39 | 4.76E-03 |
| CCDC85A | -6.52 | 3.65E-07 |
| TENM2 | -6.53 | 6.09E-03 |
| NEFM | -6.65 | 2.53E-03 |
| RYR2 | -7.02 | 3.65E-07 |

**Supp Table 3.** Top most differently expressed genes (DEGs) from total RNA sequencing on human GBM patient samples. The expression level changes of DEGs in GBM versus control samples were expressed as the absolute log-transformed fold change (logFC).

.

**
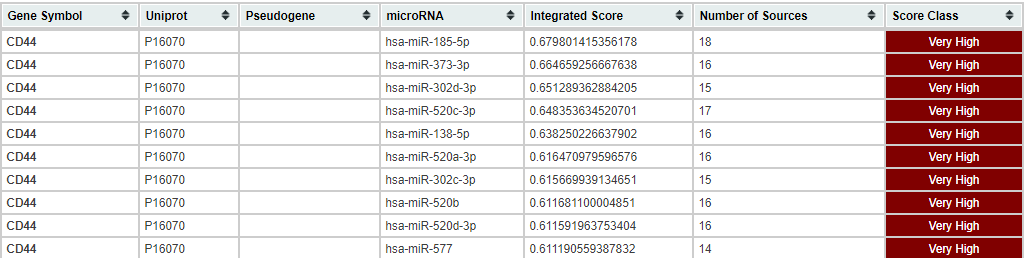
**

**Supp Table 4.** Top miRNAs targeting the 3’ UTR of CD44 as predicted by prediction software, mirDIP (microRNA Data Integration Portal). miR-138 was predicted as the fifth out of top 10 miRNAs to target CD44.

**
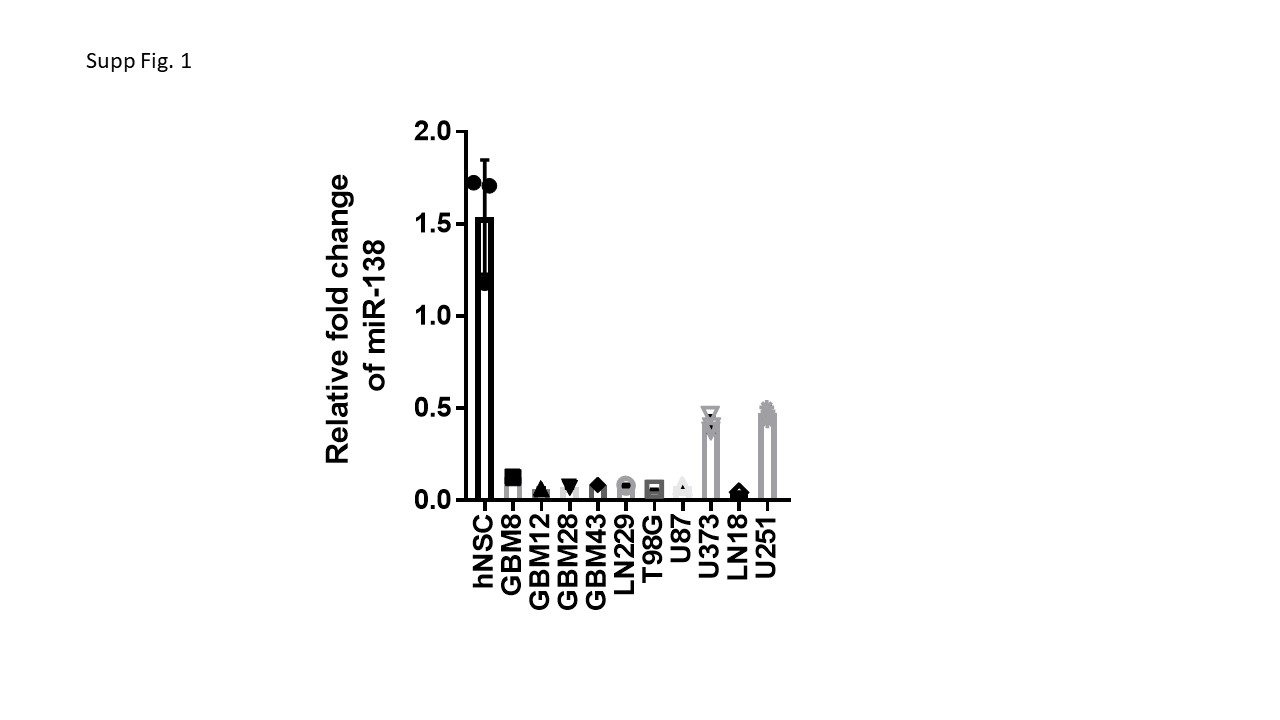
**

**Figure S1.** Relative fold change of miR-138 expression was observed lower in 4 patient-derived primary GBM cells (GBM8 (0.1262 ± 0.004762), GBM12 (0.06386 ± 0.004912), GBM28 (0.07143 ± 0.004731), GBM43 (0.08453 ± 0.002392)) and 6 glioma cell lines (LN229 (0.081 ± 0.005741), T98G (0.06172 ± 0.001572), U87 (0.07579 ± 0.002727), U373 (0.4155 ± 0.04372), LN18 (0.04435 ± 0.001073) and U251 (0.4743 ± 0.03747)) compared to human neural stem cell (hNSC (1.537 ± 0.3098) as a negative control. The expression level of miR-138 was measured by qRT-PCR using TaqMan individual miRNA assay.

**
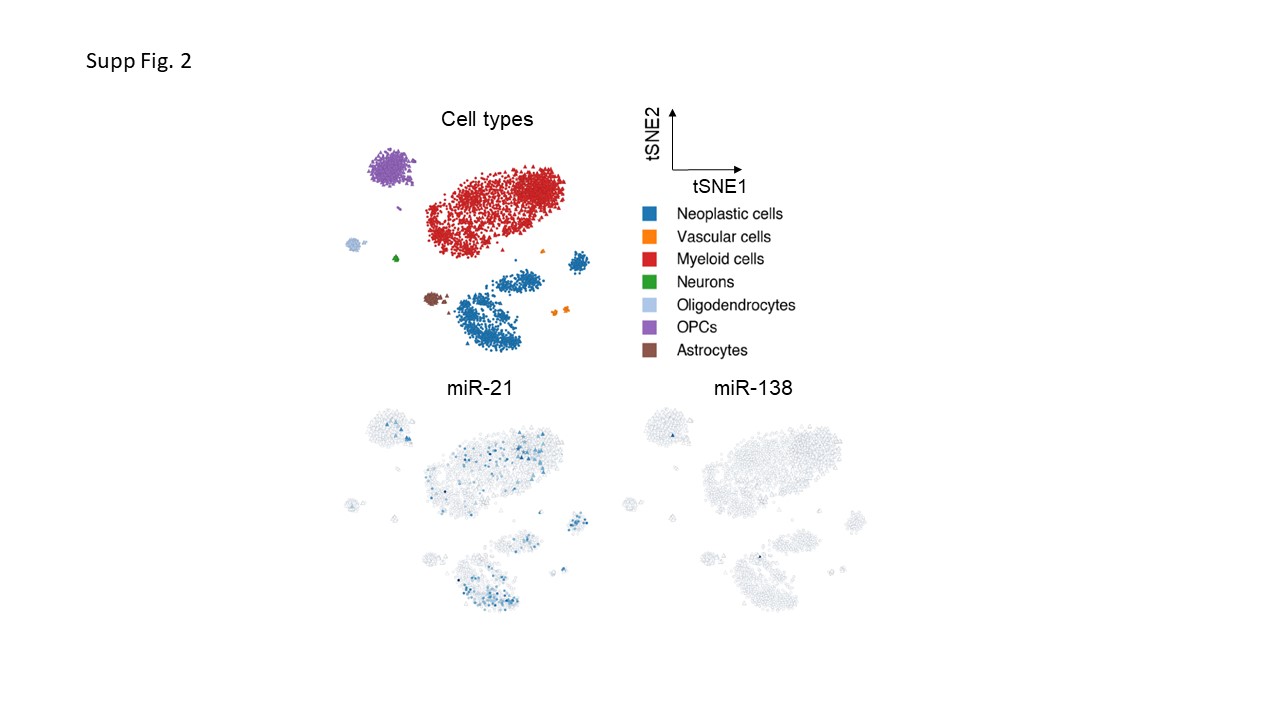
**

**Figure S2.** Expression level of miR-138 in GBM as revealed by Single cell RNA sequencing (scRNA-seq) (GBMSeq database: <http://www.gbmseq.org/>). tSNE plot showed the expression level of miR-138 barely observed in most cell types of GBM tumor, while overexpression of miR-21 was detected from neoplastic cells.

**
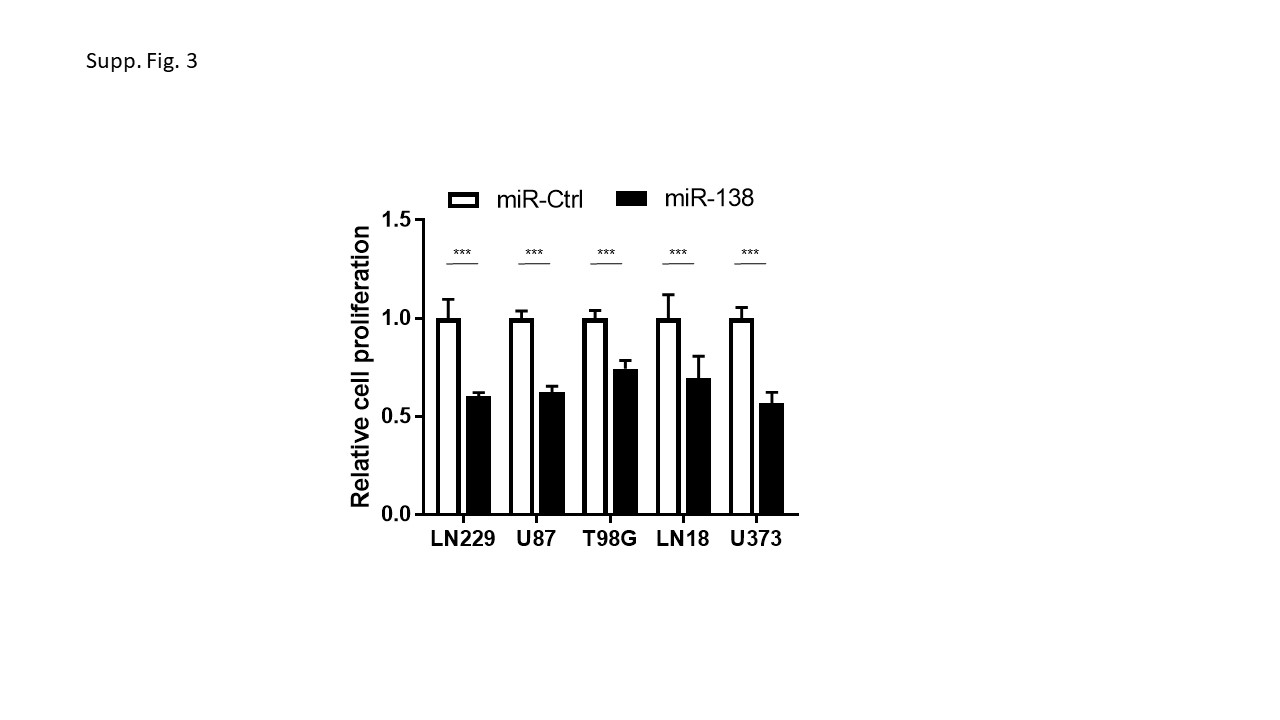
**

**Figure S3.** miR-138 restoration significantly inhibited the cell proliferation of representative GBM cells lines (LN229, U87, T98G, LN18 and U373). GBM cell lines were transfected with miR-138 or miR-Ctrl for 96 days. Cell viability was measured by CellTiter-Glo Luminescent Cell Viability Assay (n=3). All error bars indicate standard deviations, and the *p*-values were determined by two-tailed student *t*-test. ****p* < 0.001.

**
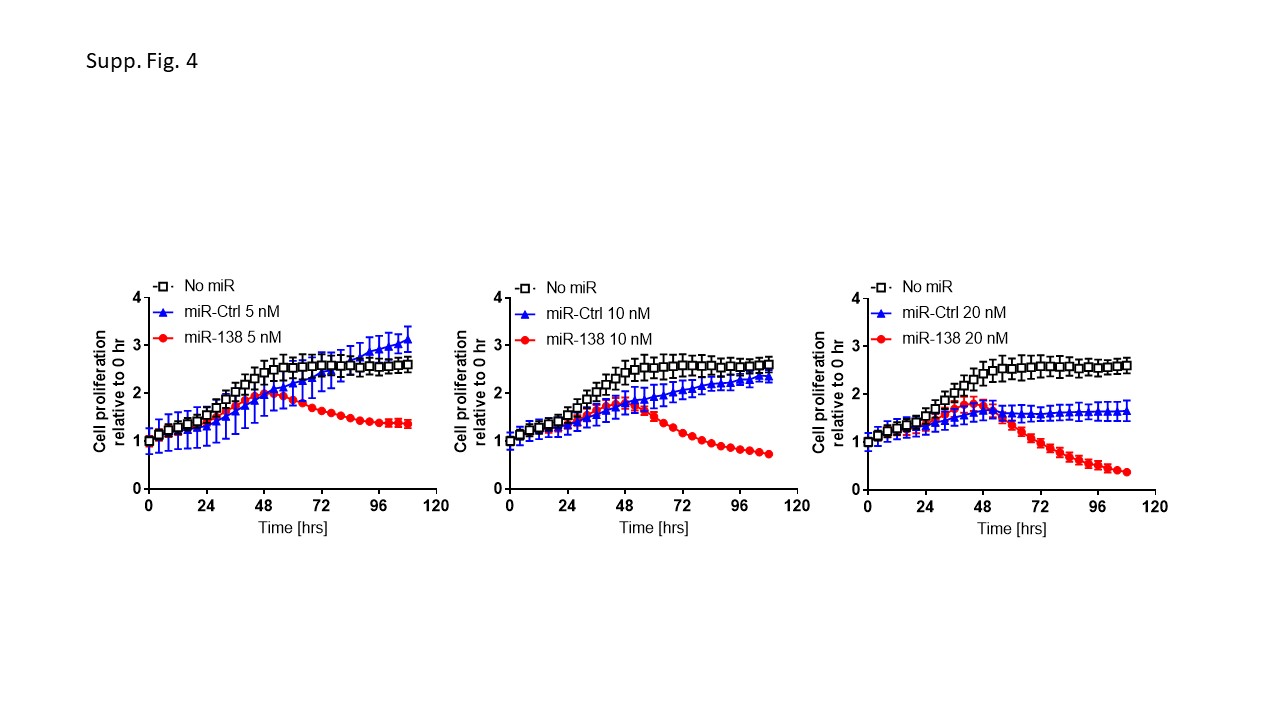
**

**Figure S4.** miR-138 restoration in GBM cells inhibited cell proliferation in concentration-dependent manner. Cell proliferation was analyzed by fluorescence live cell imaging every four hours on GBM12-RFP cells after transfection with 5, 10 or 20 nM of miR-138 or miR-Ctrl. RFP positive cells were referred to viable GBM cells (n=3). All error bars indicate standard deviations, and the *p*-values were determined by two-tailed student *t*-test. ****p* < 0.001.

**
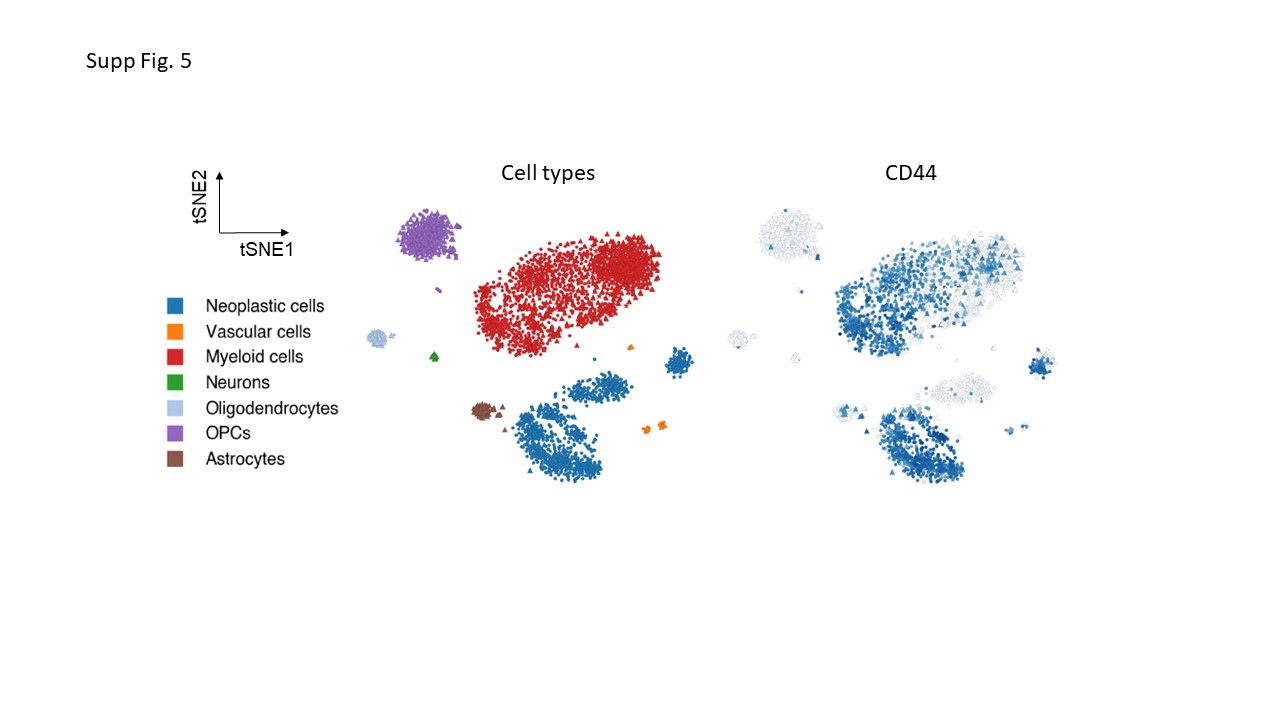
**

**Figure S5.** Expression level of *CD44* in GBM as revealed by single cell RNA sequencing (GBMSeq database: <http://www.gbmseq.org/>). tSNE plot showed that CD44 is heavily overexpressed in neoplastic cells.

**
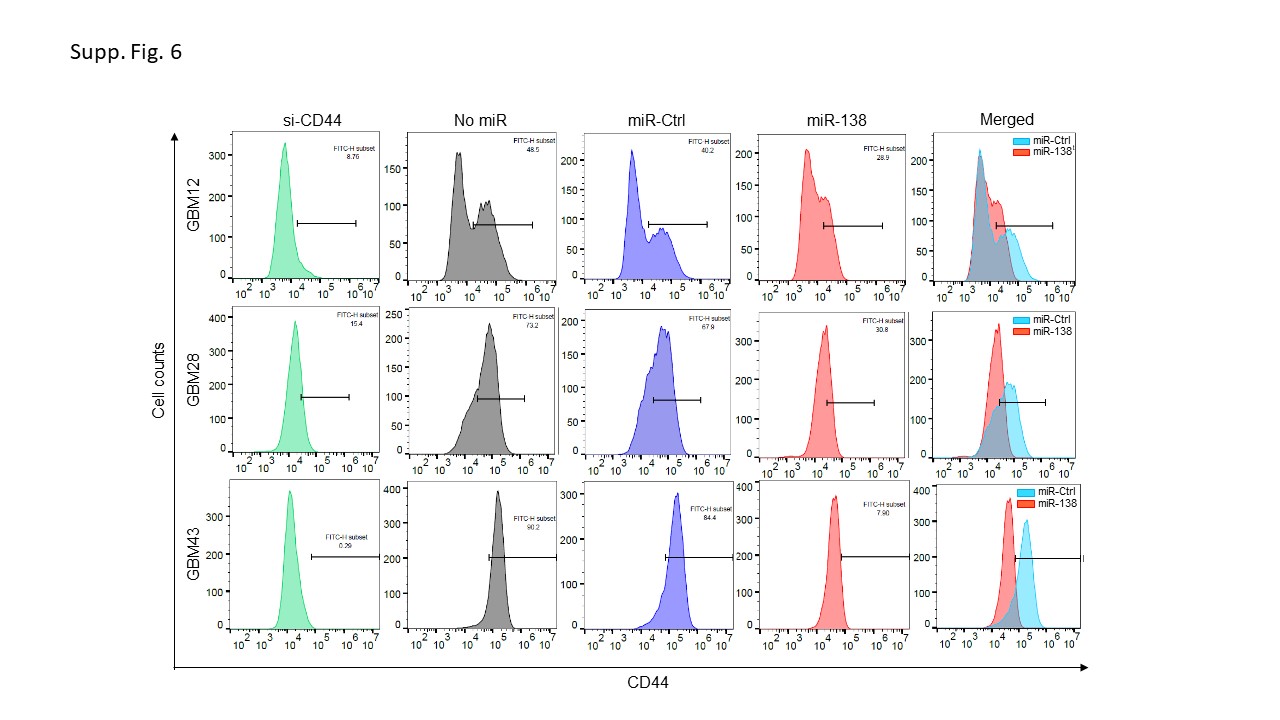
**

**Figure S6.** Representative flow cytometry images to detect CD44 positive cells from GBM cells (GBM12, GBM28 or GBM43) transfected with miR-138. Transiently transfected GBM cells with miR-138 mimics or miR-Ctrl were stained with FITC-labeled anti-CD44 antibodies and analyzed. GBM cells transfected with siRNAs against CD44 were used to gate FITC-CD44 negative cell population (n=3).

**
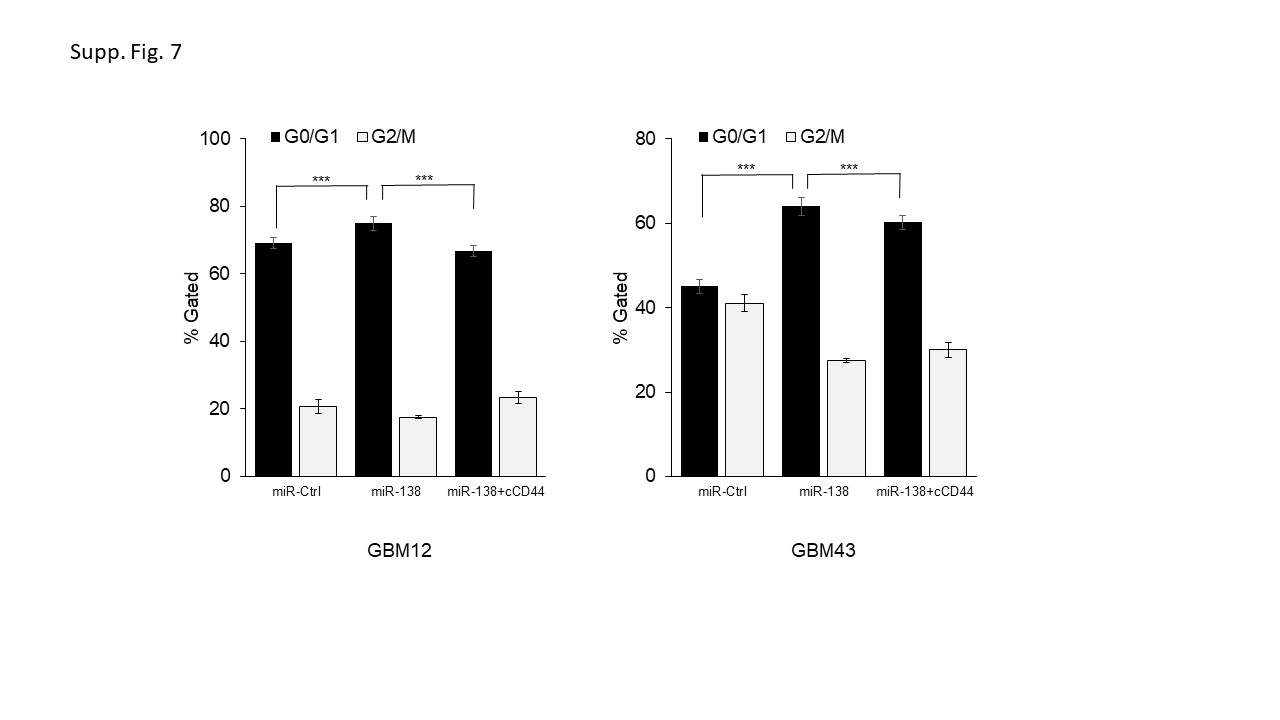
**

**Figure S7.** Cell cycle analysis by flow cytometry on GBM12 or GBM43 cells after 4 days of transient transfection of miR-138 or miR-Ctrl. Comparison of cell populations between G0/G1 and G2/M phases were expressed as %gated cells to total cell populations. Arrested cell cycle at G0/G1 phase by miR-138 was partially reversed by ectopic overexpression of CD44. All error bars indicate standard deviations, and the *p*-values were determined by two-tailed student *t*-test. ****p* < 0.001.

**
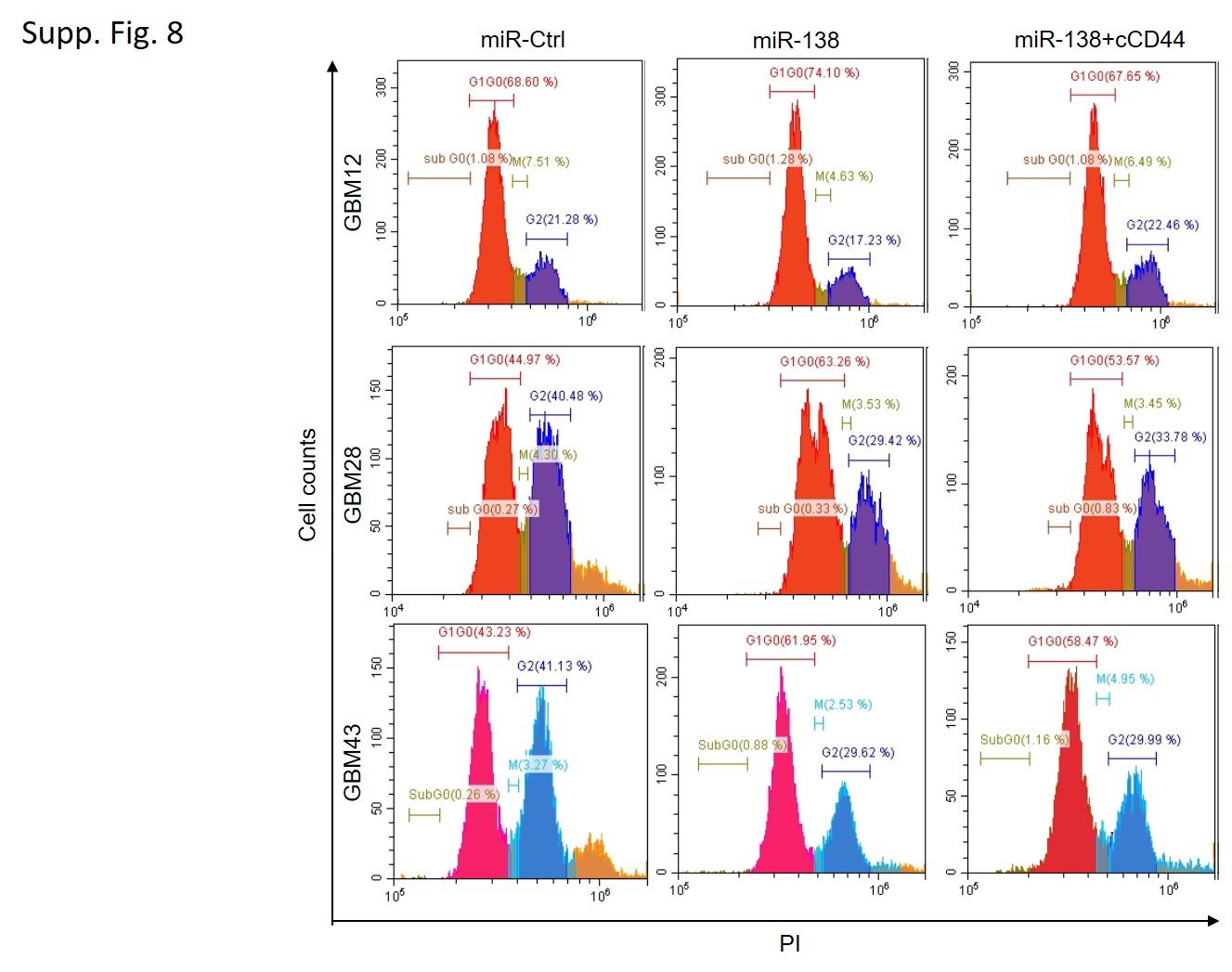
**

**Figure S8.** Representative cytograms from cell cycle analysis by flow cytometry on GBM12, GBM28 or GBM43 cells after 4 days of transient transfection of miR-138 or miR-Ctrl. Comparison of cell populations between G0/G1 and G2/M phases were expressed as %gated cells to total cell populations. Arrested cell cycle at G0/G1 phase by miR-138 was partially reversed by ectopic overexpression of CD44.

**
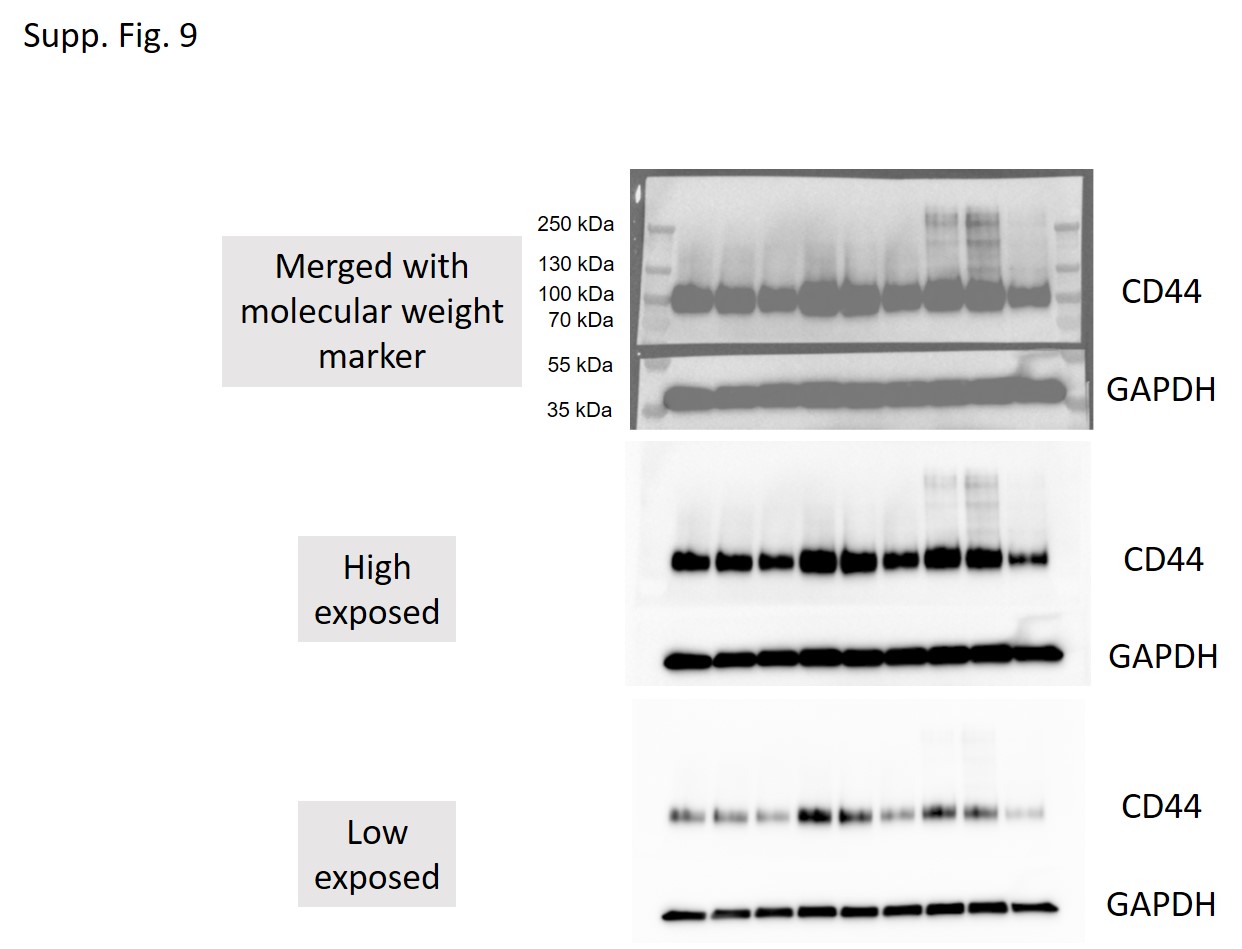
**

**Figure S9.** Original full-length image of western blotting analysis used in the Figure 3E. The immunoreactive bands on nitrocellulose membranes were visualized with enhanced chemiluminescence (ECL) (GE Healthcare, Piscataway, NJ, USA) using ChemiDoc MP (Bio-Rad, Hercules, CA, USA). The bands corresponding to CD44 or GAPDH were cropped to be used in the main figure. The positions of molecular weight bands visualized separately under bright filed mode was merged into chemiluminescence images. High and low exposure images were provided.

**
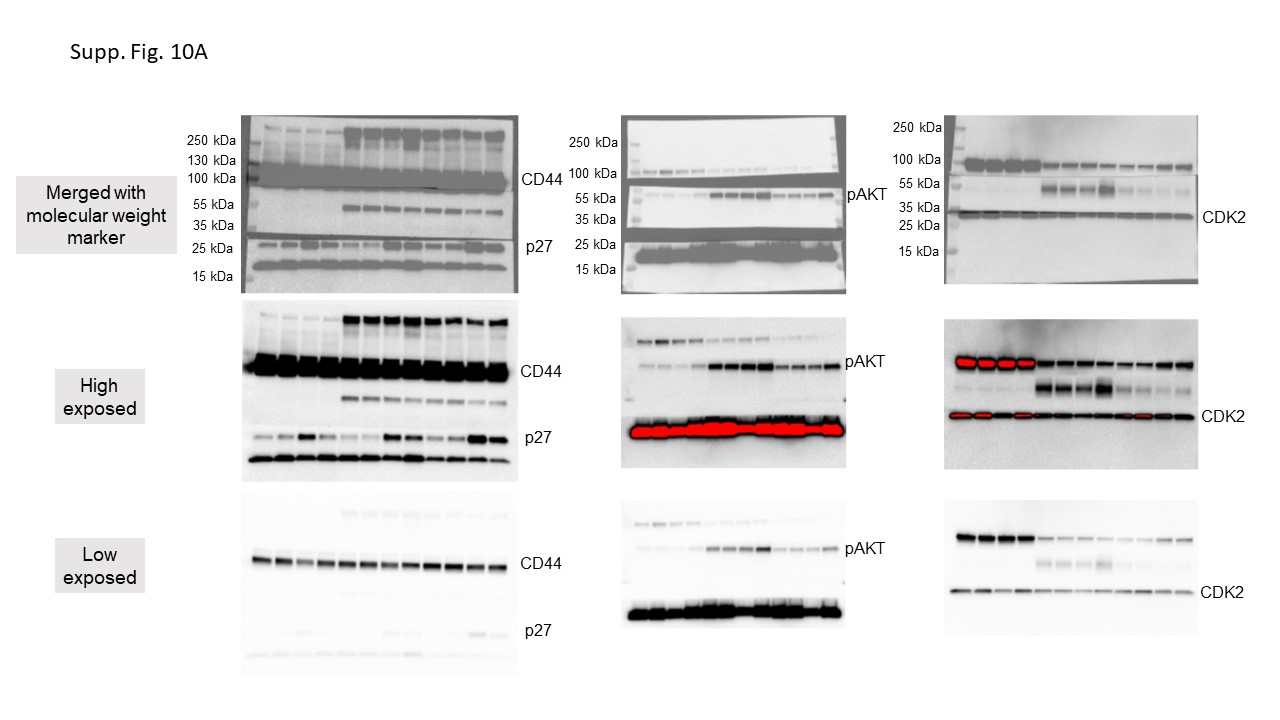

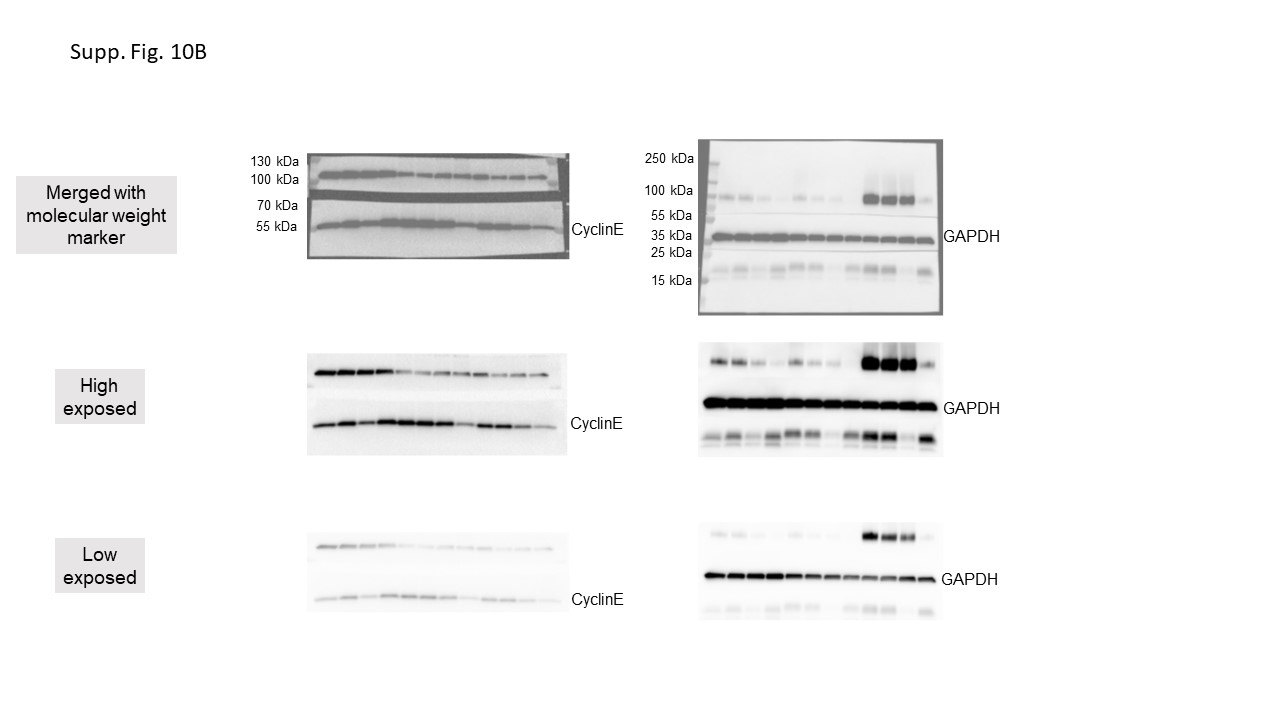
**

**Figure S10A and S10B.** Original full-length image of western blotting analysis used in the Figure 4C. The immunoreactive bands on nitrocellulose membranes were visualized with enhanced chemiluminescence (ECL) (GE Healthcare, Piscataway, NJ, USA) using ChemiDoc MP (Bio-Rad, Hercules, CA, USA). The bands corresponding to CD44, p-AKT, CDK2, p27, Cyclin E or GAPDH were cropped to be used in the main figure. The positions of molecular weight bands visualized separately under bright filed mode was merged into chemiluminescence images. High and low exposure images were provided.


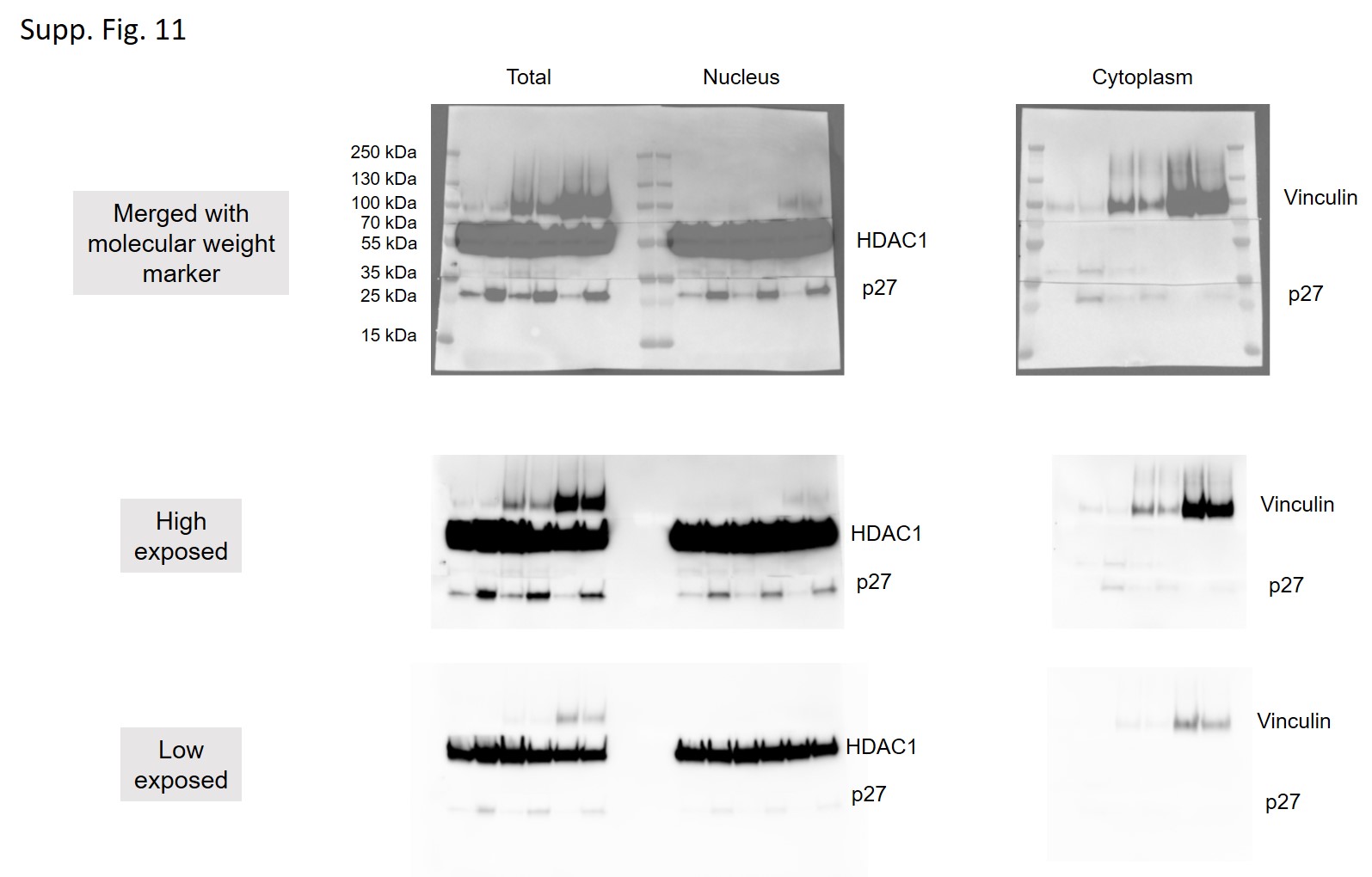


**Figure S11.** Original full-length image of western blotting analysis used in the Figure 4D. The immunoreactive bands on nitrocellulose membranes were visualized with enhanced chemiluminescence (ECL) (GE Healthcare, Piscataway, NJ, USA) using ChemiDoc MP (Bio-Rad, Hercules, CA, USA). The bands corresponding to p27, HDAC1 or Vinculin were cropped to be used in the main figure. The positions of molecular weight bands visualized separately under bright filed mode was merged into chemiluminescence images. High and low exposure images were provided.

**
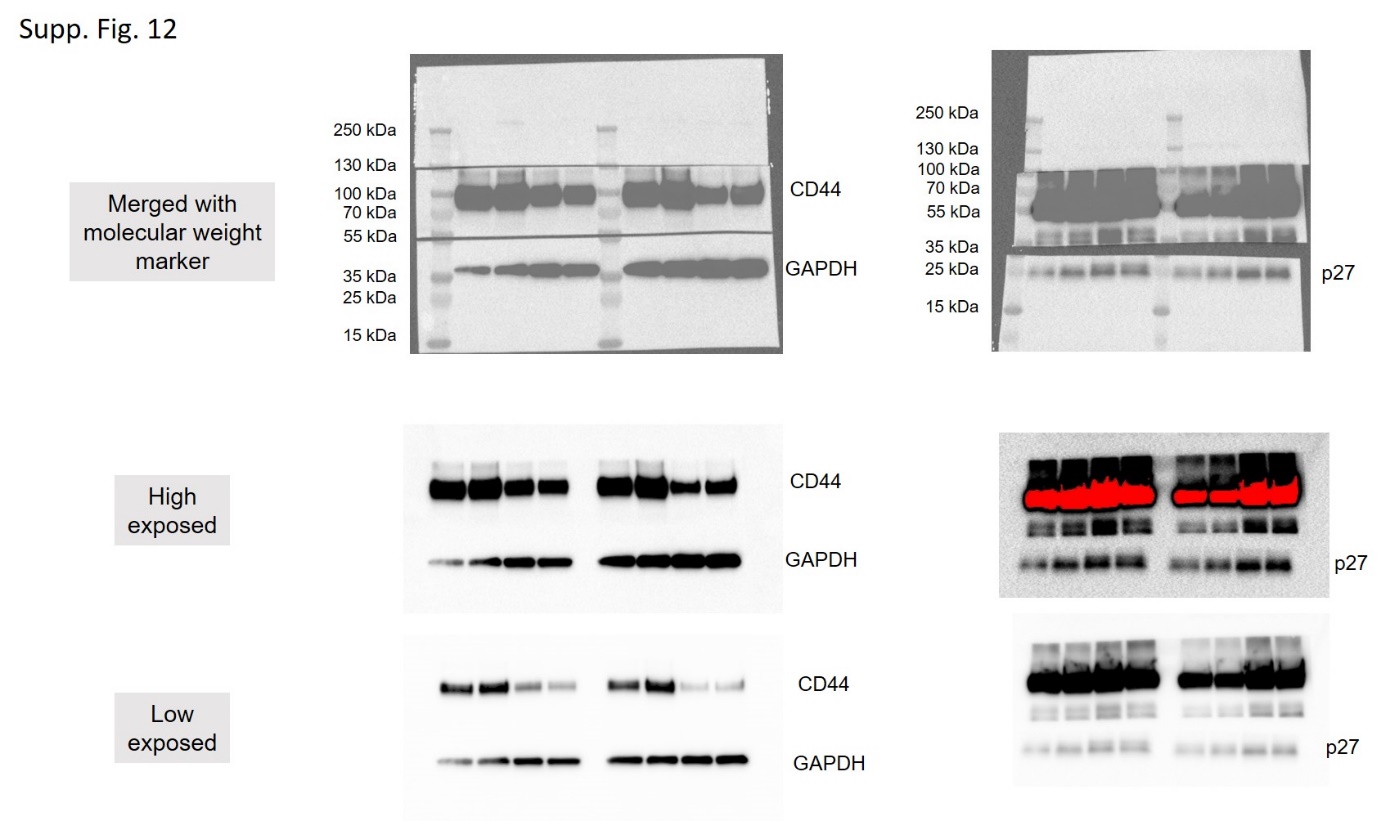
**

**Figure S12.** Original full-length image of western blotting analysis used in the Figure 5B. The immunoreactive bands on nitrocellulose membranes were visualized with enhanced chemiluminescence (ECL) (GE Healthcare, Piscataway, NJ, USA) using ChemiDoc MP (Bio-Rad, Hercules, CA, USA). The bands corresponding to CD44, p27 or GAPDH were cropped to be used in the main figure. The positions of molecular weight bands visualized separately under bright filed mode was merged into chemiluminescence images. High and low exposure images were provided.
